# Theoretical insight on the treatment of β-hexachlorocyclohexane waste through alkaline dehydrochlorination

**DOI:** 10.1038/s41598-021-88060-7

**Published:** 2021-04-22

**Authors:** Alicia Bescós, Clara I. Herrerías, Zoel Hormigón, José Antonio Mayoral, Luis Salvatella

**Affiliations:** 1 Instituto de Síntesis Química y Catálisis Homogénea (ISQCH), CSIC-Universidad de Zaragoza, Pedro Cerbuna 12, 50009 Zaragoza, Spain; 2 Present Address: Instituto de Tecnología Química (ITQ-CSIC), Avenida de los Naranjos s/n, 46022 Valencia, Spain

**Keywords:** Pollution remediation, Reaction mechanisms

## Abstract

The occurrence of 4.8–7.2 million tons of hexachlorocyclohexane (HCH) isomers stocked in dumpsites around the world constitutes a huge environmental and economical challenge because of their toxicity and persistence. Alkaline treatment of an HCH mixture in a dehydrochlorination reaction is hampered by the low reactivity of the β-HCH isomer (HCl elimination unavoidably occurring through *syn* H–C–C–Cl arrangements). More intriguingly, the preferential formation of 1,2,4-trichlorobenzene in the β-HCH dehydrochlorination reaction (despite the larger thermodynamical stability of the 1,3,5-isomer) has remained unexplained up to now, though several kinetic studies had been reported. In this paper, we firstly show a detailed Density Functional study on all paths for the hydroxide anion-induced elimination of β-HCH through a three-stage reaction mechanism (involving two types of reaction intermediates). We have now demonstrated that the first reaction intermediate can follow several alternative paths, the preferred route involving abstraction of the most acidic allylic hydrogen which leads to a second reaction intermediate yielding only 1,2,4-trichlorobenzene as the final reaction product. Our theoretical results allow explaining the available experimental data on the β-HCH dehydrochlorination reaction (rate-determining step, regioselectivity, instability of some reaction intermediates).

## Introduction

The occurrence of many uncontrolled stockpiles containing large quantities of Persistent Organic Pollutants (POPs) constitutes a major environmental, economical challenge to be solved^1,2^. Interestingly, most of such waste is derived from the 1,2,3,4,5,6-hexachlorocyclohexane (HCH) production by means of benzene photochlorination. Between 4.8 and 7.2 million tons of HCH waste are estimated to have been produced and stocked around the world^3^.

**Figure 1:**
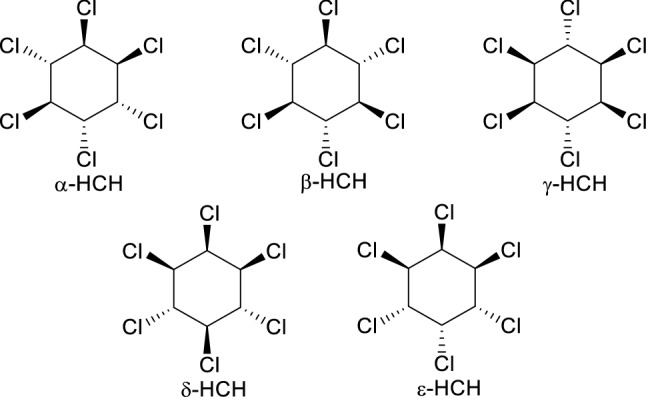
Major HCH diastereomers.

The raw product from benzene photochlorination is mostly composed of a mixture of HCH diastereomers named by Greek letters (55–80% α, 5–14% β, 8–15% γ, 2–16% δ, 3–5% ε, see Fig. 1)^4^. In the 1940s and 50s, the technical HCH mixture as a whole was commercialized as an insecticide. Since insecticidal properties are indeed only due to γ-HCH (lindane), the industrial process was subsequently modified to extract that stereoisomer, whereas the remaining isomers (as well as non-extracted γ-HCH) were deposed in dumpsites close to the production plants, which have become serious pollution foci^4^.

Despite the apparent similitude between HCH isomers, β-HCH shows a particular chemical behavior. Because of the all-*trans* arrangement of chlorine atoms in β-HCH (all H–C–C–Cl sequences showing *cis* dispositions), elimination is significantly hampered. Thus, Cristol et al. showed that β-HCH reacts with hydroxide ion in an ethanol/water mixture at 30 ºC much more slowly than other isomers (from 7000 to 24,000 times)^5^.

Significant environmental differences between HCH isomers can accordingly be found^6^. Thus, α-HCH and γ-HCH are slowly hydrolyzed in seawater (hydrolytic half-lives are 26 and 42 a, respectively)^7^ whereas a negligible reactivity of β-HCH can be inferred from Cristol data (half-life = 38–96 Ga). High persistence of β-HCH is also found for bacterial degradation since LinA-type enzymes (using an E2 path)^8^ degrade α-, γ- and δ-HCH, but not the β isomer^9^. As a consequence of the scarcity of natural degradation mechanisms, β-HCH is becoming the predominant isomer in the environment^10^ as observed in the Baltic Sea water^11^ and human fatty tissues^12^.

Because of their suspected carcinogenic, persistent, bioaccumulative, and endocrine-disrupting properties, α-, β-, and γ-HCH isomers were included in Annex A of the Stockholm Convention on Persistent Organic Pollutants^13^ thus compelling the signing countries to eliminate releases of such compounds. Evidently, the best strategy to avoid accidental HCH emissions should include the transformation of HCH stockpiles into innocuous materials^3^.

Inexpensive safe treatment of large quantities of HCH waste may be envisaged through dehydrochlorination reaction with sodium hydroxide. However, obtaining quantitative yields in the alkaline treatment of an HCH mixture is seriously hampered by the low reactivity of the β-isomer. Reaction mixtures including significant concentrations of β-HCH should not be acceptable.

As a further challenge, the alkali elimination of HCH isomers (including β-HCH) preferentially leads to 1,2,4-trichlorobenzene^14^ despite the larger stability of 1,3,5-isomer^15^. No satisfactory explanation has been given up to now to such a paradoxical result.

In this work, we study the complete mechanism for the β-HCH + hydroxide anion reaction using Density-Functional calculations. We hope that a better mechanistic insight on that process can help to a rational design of HCH waste treatment.

## Results and discussion

### Elimination pathways

**Figure 2:**
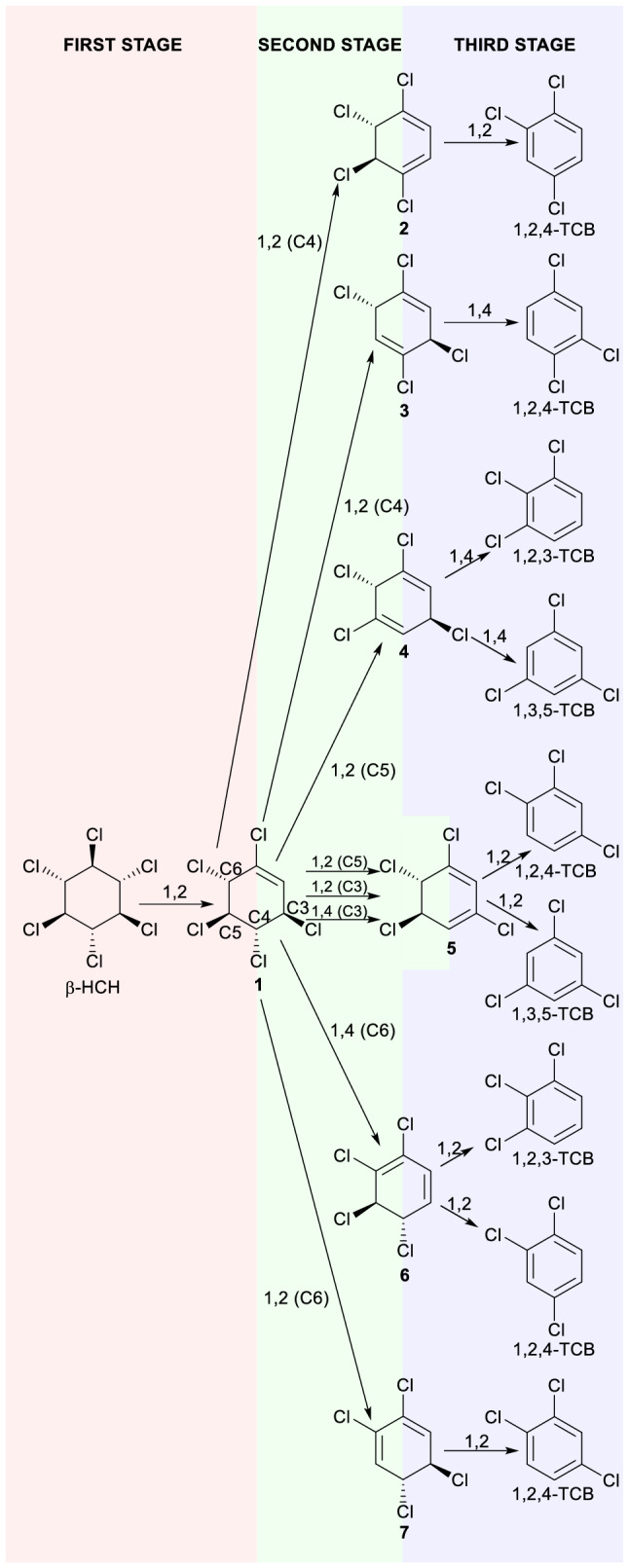
β-HCH dehydrochlorination reaction pathways indicating the regiochemistry (1,2 vs. 1,4) and the carbon undergoing deprotonation (in parentheses, if necessary) of every elimination step.

The β-HCH dehydrochlorination reaction takes place through three stages (Fig. 2). The first reaction stage corresponds to β-HCH elimination yielding *rel*-(3*R*,4*S*,5*R*,6*S*)-1,3,4,5,6-pentachlorocyclohexene (**1**). The Gibbs free energy of activation (starting from the β-HCH + HO^−^ pre-reactive complex) for the first stage was firstly studied by using different theoretical levels in gas phase and two continuum models. Results on the activation barrier (see Table S1) are consistent with well-known trends for theoretical levels (exaggeration by HF^16^, underestimation for BLYP^17^, similar results for B3LYP and M06-2X^18^) and continuum models (lower activation barriers in gas phase^19^, close results for IEFPCM and CPCM models^20^). As a consequence, CPCM(water)/M06-2X/6-311++G(d,p) calculations are only considered along the paper.

The activation barrier for the first reaction stage (9.3 kJ mol^−1^) is much lower than the experimental value for the β-HCH + sodium hydroxide reaction in ethanol at 30 ºC (129.8 kJ/mol)^21^, consistently with the overestimated reactivity of anions by using continuum solvent models^22^. Nevertheless, the occurrence of a positive activation barrier for the first reaction stage for β-HCH (in contrast with the lack of calculated TSs for other isomers) is consistent with the well-known aversion for the *syn* H–C–C–Cl arrangement in elimination reactions^14^.

In the second reaction stage, eight dehydrochlorination reaction paths starting from **1** (three of them leading to **5** as a common product) to yield tetrachlorocyclohexadienes **2**–**7** can be envisaged. Both 1,2 (involving two contiguous sp^3^ carbons) and 1,4 (involving both sp^3^ allylic carbons) regiochemistries are possible.

Pathways for dehydrochlorination of **1** leading to tetrachlorocyclohexadienes **2**–**6** showed positive activation Gibbs free energies. Routes involving the removal of allylic hydrogens from C3 and C6 (4.2–9.6 kJ mol^−1^) are clearly preferred over those implying non-allylic atoms C4 and C5 (33.4–34.8 kJ mol^−1^), in agreement with the relative acidity of allylic hydrogens. When routes leading to conjugated tetrachlorocyclohexadienes are compared, a preference for the formation of **5** (involving allylic proton removal) relative to **2** (implying non-allylic hydrogen abstraction) by a 200,000:1 ratio (according to calculated relative rate constants^23^) is found.

All attempts to locate transition states for the transformations of **1** into non-conjugated tetrachlorocyclohexadienes (**3** and **4**) yielded instead structures leading to the favored conjugated isomers.

No TS could be located for the **1** → **7** transformation using the M06-2X or the HF level (typically overestimating activation barriers^16^) since a monotonic energy descent for the **1** + HO^−^ approximation leading to **7** is found instead. A strong preference for the formation of **7** as a key intermediate in the β-HCH elimination, even larger than that corresponding for the formation of the other conjugated tetrachlorocyclohexadienes, can thus be inferred.

**Figure 3:**
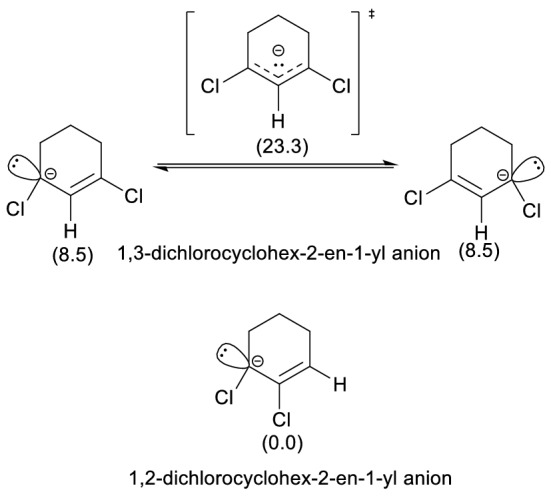
1,3- and 1,2-Dichlorocyclohex-2-en-1-yl carbanions. Calculated relative Gibbs free energies (kJ mol^−1^) are shown in parentheses.

To ascertain the source of the strong preference of **1** for the formation of **7**, calculations were carried out on 1,3- and 1,2-dichlorocyclohex-2-en-1-yl carbanions (see Fig. 3), lacking additional C_sp_^3^-bound chlorines (thus avoiding the spontaneous chloride anion leave). Interestingly, non-planar π-allyl structures were found in both cases, similarly to 1-fluoro or 1-hydroxy substituted allyl anions through HF/6-31+G(d) calculations^24^. Such a geometrical feature can be attributed to the preference for the pyramidal geometry of the carbanionic carbon (lone pair showing a 22.13% s+77.19% p hybridization, according to NBO calculations) as a consequence of the C–Cl bond polarity^25^, analogously to the strong predilection for pyramidal geometry in NF_3_^26^.

For 1,3-dichlorocyclohex-2-en-1-yl anion, two enantiomeric minima can interconvert through a delocalized TS, thus indicating a stabilization through localization (16.5 kJ mol^−1^ in electronic energy; 14.8 kJ mol^−1^ in Gibbs free energy) in contrast with the localization destabilization of the non-substituted allyl anion^27^. An energy minimum was found for the localized 1,2-dichlorocyclohex-2-en-1-yl carbanion, but not for the alternative localized allylic system (2,3-dichlorocyclohex-2-en-1-yl anion).

Calculations show larger stability for 1,2-dichlorocyclohex-2-en-1-yl carbanion relative to the 1,3-isomeric anion (8.0 kJ mol^−1^ in electronic energy; 8.5 kJ mol^−1^ in Gibbs free energy). These results show that the stabilization of an allylic carbanion by a chlorine substituent is larger on the central position than that on the extremal site, consistently with the larger gas phase acidity of 2-chloropropene^28^ relative to 1-chloropropene^24^.

Calculations show larger stability for 1,2-dichlorocyclohex-2-en-1-yl carbanion relative to the 1,3-isomeric anion (8.0 kJ mol^−1^ in electronic energy; 8.5 kJ mol^−1^ in Gibbs free energy). These results show that a chlorine in the central position increases the stability of the allylic carbanion more than a chlorine in the extremal site, which is consistent with the fact that 2-chloropropene is more acidic than 1-chloropropene in gas phase^24^.

In other words, chloroalkenes show a preference for proton removal from the CH group next to the chlorine-bearing vinylic carbon rather than that contiguous to hydrogen-bearing vinylic carbon.

The extension of such a conclusion to reaction intermediate **1** allows understanding the preferential deprotonation from C6 rather than proton removal from C3. Although both 1,2- and 1,4-elimination regiochemistries (leading, respectively, to **7** and **6**) can be envisaged, a preference for the formation of **7** in the second stage is finally found.

The third reaction stage corresponds to the elimination of tetrachlorocyclohexadienes **2**–**7** yielding trichlorobenzene (TCB) isomers. Two alternative dehydrochlorination paths (or one, if equivalent) are possible starting from every tetrachlorocyclohexadiene (involving the chloride anion leave from each carbon) leading to two (or one) trichlorobenzene(s). Very low or negative activation Gibbs free energies (between −3.2 kJ mol^−1^ and +3.8 kJ mol^−1^) are found for the third stage involving the elimination of intermediates **2**–**7** (monotonic energy descents were indeed found for both transformations **3** → 1,2,4-TCB and **4** → 1,3,5-TCB). Such results can be attributed to the aromaticity emergence as the driving force of the third stage. The high decomposition rate of tetrachlorocyclohexadienes allows explaining the composition of Dense Nonaqueous Phase Liquid (DNAPL) formed in HCH dumpsites, typically rich in hexachlorocyclohexanes, pentachlorocyclohexenes, and trichlorobenzenes, but lacking tetrachlorocyclohexadienes^29^.

The preferred reaction pathway involves the sequence β-HCH → **1** → **7** → 1,2,4-TCB where the first stage corresponds to the rate-limiting step, as typically found in kinetic studies^5,14^. A more detailed description of such a preferred path is gathered below.

### Preferred reaction path

**Figure 4:**
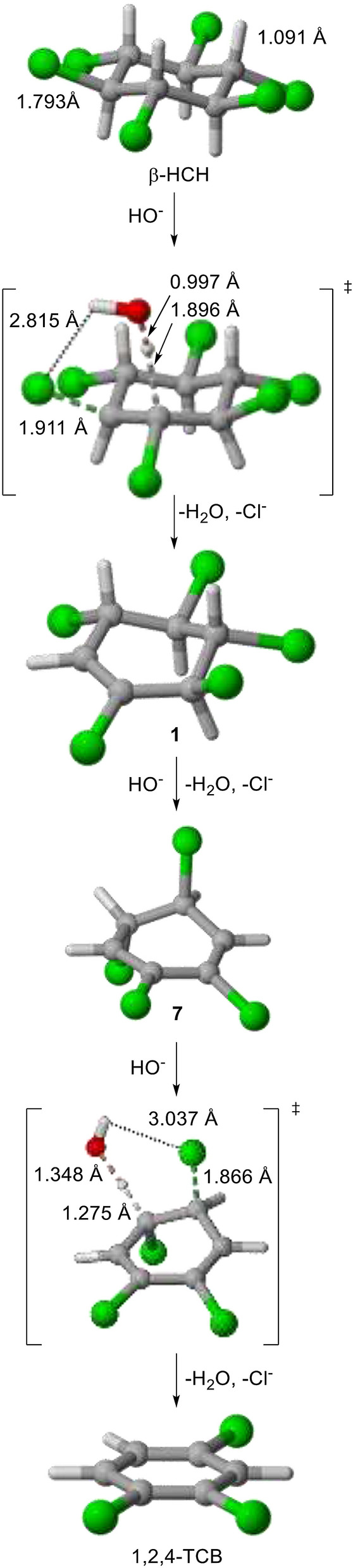
Preferred reaction path for the complete β-HCH + hydroxide anion elimination. Three-dimensional structures were generated using CYLView^44^.

Calculated reaction intermedia and TSs involved in the most favored pathway for the complete elimination reaction of β-HCH is shown in Fig. 4. A preference for the all-equatorial conformation is found for β-HCH.

The reaction of β-HCH with hydroxide anion leads to a very asynchronous TS for the first reaction stage, the C–H bond being essentially broken (1.896 Å), whereas the C–Cl bond is almost intact (1.911 Å). High asynchronicity for *syn* E2 TSs (in comparison with *anti* structures) has also been reported in theoretical studies on reactions of fluoride anion with fluoroethane^30^ and iodoethane^31^. The high asynchronicity of a *syn* 1,2-elimination TSs (involving a very advanced proton removal and a very incipient chloride anion leave) allows explaining the strong influence of proton acidity on activation barriers. A chair conformation is adopted by the cyclohexane ring in the TS for the first elimination stage, in contrast with Hine's proposal on a boat form^21^. On the other hand, the water molecule (formed by proton removal by hydroxide anion) is bound to the leaving chloride anion through electrostatic interactions (2.815 Å).

Reaction intermediate **1** is obtained in the first reaction stage. Such species can undergo a new reaction with hydroxide anion through different paths, though the preferred route yields reaction intermediate **7**. Instead, intermediate **7** can only undergo a new HCl elimination through two equivalent paths.

Comparison between TSs for β-HCH → **1** and **7** → 1,2,4-TCB transformations shows a more reactant-like geometry for the latter (breaking C···H bond length: 1.896 Å vs. 1.275 Å, respectively), in agreement with the Hammond postulate because of more negative reaction energy due to the trichlobenzene aromaticity emergence. A very incipient water-assistance for the chloride anion (Cl···H distance = 3.037 Å) is also found.

The preferential formation of 1,2,4-TCB as the major reaction product has been usually observed in the elimination of β-HCH (as well as other isomers)^14^ despite the larger stability of the 1,3,5-isomer. Such a result was attributed in a study on γ-HCH (lindane) dehydrochlorination to the occurrence of two conformers of *rel*-(3*R*,4*S*,5*S*,6*R*)-1,3,4,5,6-pentachlorocyclohexene (a diastereomer of **1**) as the first reaction intermediate, leading to two different regioisomers of tetrachlorocyclohexa-1,4-diene, which finally afford 1,2,3- and 1,3,5- or, preferentially, 1,2,4-TCB^32^. In contrast, our study on β-HCH elimination allows attributing the overall reaction selectivity to the higher acidity of a CH allylic group of the first reaction intermediate **1** which leads to the preferential formation of **7** (inexorably yielding 1,2,4-TCB).

## Conclusion

In this theoretical study, the full mechanism on the β-HCH elimination reaction by hydroxide anion has been firstly reported. Results allow obtaining an insight into the three-stage reaction mechanism. The first stage involves the E2 elimination of β-HCH to yield **1**. Although several pathways are possible for the subsequent **1** elimination in the second stage, the preferred path shows the proton removal from C6 (allylic carbon next to the chlorine-bearing vinylic carbon) to yield **7**. The subsequent elimination from **7** in the third stage can only yield 1,2,4-TCB. These results allow explaining experimental data showing that the rate-determining step corresponds to the first stage as well as the preferential formation of 1,2,4-TCB. We think that this new detailed insight on the reaction mechanism of the β-HCH dehydrochlorination reaction can be helpful in the rational design of the treatment for HCH waste stockpiles (e. g., involving a specific β-HCH treatment after separation by precipitation)^33^.

### Computational methods

Calculations were carried out by using the Gaussian 09 package (version A.02)^34^. The activation barrier for the first stage (rate-controlling step) was studied by using Hartree-Fock^35^, BLYP^36,37^, B3LYP^36–38^, and M06-2X^39^ theoretical levels with the 6-311++G(d,p) basis set for all atoms through gas-phase as well as IEFPCM and CPCM (both involving water as an implicit solvent) media models. Instead, the full reaction mechanism (including all plausible conformations) was studied by means of the M06-2X functional since M06-2X/6-311++G(d,p) calculations had been successfully applied to some related studies, such as halogenated cyclohexanes^40^, cyclohexane puckering^41^ and E2 elimination (both *anti* and *syn*) reaction^42^. Calculations were studied through CPCM media model. All energy minima were identified by the lack of imaginary analytical frequencies, whereas all TSs were characterized by the occurrence of one imaginary frequency as well as Intrinsic Reaction Coordinate calculations linking the structures with the expected energy minima. 1,3-Dichlorocyclohex-2-en-1-yl anion was studied by means of Natural Bond Orbital (NBO) Analysis^43^ as implemented in Gaussian09. Hard data are gathered in the Electronic Supplementary Information.

## Supplementary Information

Supplementary Information
