# Supplementary material for: Theoretical insight on the treatment of β-hexachlorocyclohexane waste through alkaline dehydrochlorination
Source: Sci Rep. 2021 Apr 22;11:8777. doi: 10.1038/s41598-021-88060-7 (PMC8062475; doi:10.1038/s41598-021-88060-7)
Supplement: Supplementary file 1 — Supplementary Information [file 41598_2021_88060_MOESM1_ESM.docx]

**Supplementary Information**

**Theoretical insight on the treatment of β-hexachlorocyclohexane waste through alkaline dehydrochlorination**

**Alicia Bescós^1^, Clara I. Herrerías^1^, Zoel Hormigón^1,2^, José Antonio Mayoral^1^ & Luis Salvatella^1^**^🖂^

^1^Instituto de Síntesis Química y Catálisis Homogénea (ISQCH), CSIC-Universidad de Zaragoza, Pedro Cerbuna 12, E50009-Zaragoza, Spain. ^2^Present address: Instituto de Tecnología Química (ITQ-CSIC), Avenida de los Naranjos s/n, E46022-Valencia, Spain. ^🖂^email: lsalvate@unizar.es

**Table of contents**

| **Table S1.** Relative Gibbs free energies of activation at 25 ºC and 1 atm (kJ mol^-1^) for the β-HCH + HO^-^ reaction (starting from the pre-reactive complex) at different theoretical levels and medium models by using the 6‑311++G(d,p) basis set. | S2 |
| --- | --- |
| **Table S2.** Electronic energies and Gibbs free energies (Hartree) at 25 ºC and 1 atm of both pre-reactive complex and TS involved in the β-HCH + HO^-^ reaction at different theoretical levels and medium models by using the 6-311++G(d,p) basis set. | S3 |
| **Table S3.** Relative Gibbs free energies (kJ mol^-1^) for the structures involved in each reaction step at CPCM(water)/M06-2X/6-311++G(d,p) level.^a^ | S4 |
| **Table S4.** Electronic energies and Gibbs free energies at 25 ºC and 1 atm of computed structures at CPCM(water)/M06-2X/6-311++G(d,p) level. | S5 |
| **Table S5.** Cartesian coordinates of both pre-reactive complex and TS involved in the first stage of β-HCH elimination as calculated at different theoretical levels and medium models. | S7 |
| **Table S6.** Cartesian coordinates of all structures at CPCM(water)/M06-2X/6-311++G(d,p) level. | S17 |

**Table S1.** Relative Gibbs free energies of activation at 25 ºC and 1 atm (kJ mol^-1^) for the β-HCH + HO^-^ reaction (starting from the pre-reactive complex) at different theoretical levels and medium models by using the 6‑311++G(d,p) basis set.

| **Theoretical level** | **Medium model** | **Activation energy** |
| --- | --- | --- |
| HF | gas phase | 25.8 |
| HF | IEFPCM | 41.2 |
| HF | CPCM | 42.4 |
| BLYP | gas phase | 8.1 |
| BLYP | IEFPCM | 5.5 |
| BLYP | CPCM | 9.4 |
| B3LYP | gas phase | 4.0 |
| B3LYP | IEFPCM | 15.5 |
| B3LYP | CPCM | 15.4 |
| M062X | gas phase | 3.9 |
| M062X | IEFPCM | 11.7 |
| M062X | CPCM | 9.3 |

**Table S2.** Electronic energies and Gibbs free energies (Hartree) at 25 ºC and 1 atm of both pre-reactive complex and TS involved in the β-HCH + HO^-^ reaction at different theoretical levels and medium models by using the 6-311++G(d,p) basis set.

| **Structure** | **Theoretical level** | **Medium model** | **Electronic energy (Ha)** | **Gibbs free energy (Ha)** |
| --- | --- | --- | --- | --- |
| pre-reactive complex | HF | gas phase | -3063.27777225 | -3063.186166 |
| pre-reactive complex | HF | IEFPCM | -3063.36190867 | -3063.271818 |
| pre-reactive complex | HF | CPCM | -3063.36224780 | -3063.272747 |
| pre-reactive complex | BLYP | gas phase | -3069.33862011 | -3069.266407 |
| pre-reactive complex | BLYP | IEFPCM | -3069.41584673 | -3069.341768 |
| pre-reactive complex | BLYP | CPCM | -3069.41601372 | -3069.343290 |
| pre-reactive complex | B3LYP | gas phase | -3069.56766407 | -3069.488424 |
| pre-reactive complex | B3LYP | IEFPCM | -3069.64653648 | -3069.568193 |
| pre-reactive complex | B3LYP | CPCM | -3069.64682823 | -3069.568463 |
| pre-reactive complex | M06-2X | gas phase | -3069.28700225 | -3069.203858 |
| pre-reactive complex | M06-2X | IEFPCM | -3069.36650603 | -3069.285279 |
| pre-reactive complex | M06-2X | CPCM | -3069.36652872 | -3069.284443 |
| TS | HF | gas phase | -3063.26221329 | -3063.176346 |
| TS | HF | IEFPCM | -3063.34138934 | -3063.256140 |
| TS | HF | CPCM | -3063.34164571 | -3063.256585 |
| TS | BLYP | gas phase | -3069.33296334 | -3069.263316 |
| TS | BLYP | IEFPCM | -3069.40846528 | -3069.339687 |
| TS | BLYP | CPCM | -3069.40871672 | -3069.339725 |
| TS | B3LYP | gas phase | -3069.56004841 | -3069.486915 |
| TS | B3LYP | IEFPCM | -3069.63619297 | -3069.562283 |
| TS | B3LYP | CPCM | -3069.63643368 | -3069.562594 |
| TS | M06-2X | gas phase | -3069.28055526 | -3069.202360 |
| TS | M06-2X | IEFPCM | -3069.36043423 | -3069.280814 |
| TS | M06-2X | CPCM | -3069.36061728 | -3069.280908 |

**Table S3.** Relative Gibbs free energies relative to non-interacting reactants (not in parentheses) and relative to the most stable pre-reactive complex of the corresponding stage (in parentheses) for all structures at CPCM(water)/M06-2X/6-311++G(d,p) level (kJ mol^‑1^)^a^.

| **Stage** | **Reaction step** | **Reactants** | **Pre-reactive complex** | **TS** | **Reaction Intermediate** | **Second TS** | **Post-reactive complex** | **Products** |
| --- | --- | --- | --- | --- | --- | --- | --- | --- |
| First | β-HCH → **1**: 1,2 | 0.0  (15.5) | -20.4  (0.0) | -11.1  (9.3) |  |  | -192.5  (-177.1) | -213.2  (-197.7) |
| Second | **1** → **2**: 1,2 (C4) | -213.2  (28.3) | -241.5  (0.0) | -206.7  (34.8) |  |  | -408.7  (-167.2) | -440.3  (-198.8) |
| Second | **1** → **2**: 1,2 (C4) | -213.2  (28.3) | -241.5  (0.0) | -207.2  (34.3) | -219.9  (21.6) | -213.4  (28.1) | -393.3  (-151.8) | -440.3  (-198.8) |
| Second | **1** → **3**: 1,2 (C4) | -213.2  (28.3) | –^b^ | –^b^ |  |  | –^b^ | -431.8  (-190.3) |
| Second | **1** → **4**: 1,2 (C5) | -213.2  (28.3) | –^b^ | –^b^ |  |  | –^b^ | -433.0  (-191.5) |
| Second | **1** → **5**: 1,2 (C5) | -213.2  (28.3) | -241.5  (0.0) | -208.1  (33.4) |  |  | -389.5  (-148.0) | -438.3  (-196.8) |
| Second | **1** → **5**: 1,2 (C3) | -213.2  (28.3) | -241.5  (0.0) | -231.9  (9.6) |  |  | -397.3  (-155.9) | -438.3  (-196.8) |
| Second | **1** → **5**: 1,4 (C3) | -213.2  (28.3) | -241.5  (0.0) | -237.3  (4.2) |  |  | -375.6  (-134.1) | -438.3  (-196.8) |
| Second | **1** → **6**: 1,4 (C6) | -213.2  (28.3) | -241.5  (0.0) | -237.2  (4.3) |  |  | -372.8  (-131.3) | -414.5  (-173.0) |
| Second | **1** → **7**: 1,2 (C6) | -213.2  (28.3) | –^b^ | –^b^ |  |  | –^b^ | -428.6  (-187.1) |
| Third | **2** → 1,2,4-TCB: 1,2 | -440.3  (-5.1) | -435.2  (0.0) | -437.7  (-2.4) |  |  | -740.4  (-303.4) | -770.3  (-31.7) |
| Third | **3** → 1,2,4-TCB: 1,4 | -431.8  (3.4) | –^b^ | –^b^ |  |  | –^b^ | -770.3  (-31.7) |
| Third | **4** → 1,2,3-TCB: 1,4 | -433.0  (2.2) | -432.7  (2.6) | -431.6  (3.5) |  |  | -722.1  (-287.0) | -763.4  (-24.8) |
| Third | **4** → 1,3,5-TCB: 1,4 | -433.0  (2.2) | –^b^ | –^b^ |  |  | –^b^ | -775.6  (-37.0) |
| Third | **5** → 1,2,4-TCB: 1,2 | -438.3  (-3.0) | -432.9  (2.3) | -434.2  (1.0) |  |  | -740.4  (-303.0) | -770.3  (-31.7) |
| Third | **5** → 1,3,5-TCB: 1,2 | -438.3  (-3.0) | -432.9  (2.3) | -429.2  (6.0) |  |  | -744.7  (-309.5) | -775.6  (-37.0) |
| Third | **6** → 1,2,3-TCB: 1,2 | -414.5  (20.7) | -422.8  (12.4) | -409.4  (21.2) |  |  | -726.9  (-296.3) | -763.4  (-24.8) |
| Third | **6** → 1,2,4-TCB: 1,2 | -414.5  (20.7) | -422.8  (12.4) | -424.2  (10.9) |  |  | -745.0  (-309.8) | -770.3  (-31.7) |
| Third | **7** → 1,2,4-TCB: 1,2 | -428.6  (6.6) | -417.2  (18.1) | -418.2  (17.0) |  |  | -740.4  (-306.3) | -770.3  (-31.7) |

^a^ Free energy values of co-reactants (hydroxide anion) and co-products (water, chloride anion) have been included in the calculated energies.

^b^ Structure not located.

**Table S4.** Electronic energies and Gibbs free energies at 25 ºC and 1 atm of computed structures at CPCM(water)/M06-2X/6-311++G(d,p) level.

| **Structure** | **Electronic Energy (Ha)** | **Gibbs Free Energy (Ha)** |
| --- | --- | --- |
| β-HCH (all-equatorial conformer) | -2993.42853793 | -2993.351230 |
| β-HCH (all-axial conformer) | -2993.41583073 | -2993.339754 |
| hydroxide anion (HO^-^) | -75.91807776 | -75.925439 |
| water (H_2_O) | -76.42901303 | -76.425146 |
| chloride anion (Cl^-^) | -460.38000994 | -460.395033 |
| pre-reactive complex β-HCH →**1**: 1,2 | -3069.36138250 | -3069.282555 |
| TS β-HCH →**1**: 1,2 | -3069.36061728 | -3069.280908 |
| post-reactive complex β-HCH →**1**: 1,2 | -3069.42794522 | -3069.350002 |
| *rel*-(3*R*,4*S*,5*R*,6*S*)-1,3,4,5,6-pentachlorocyclohex-1-ene (**1**) | -2532.59917771 | -2532.537691 |
| pre-reactive complex **1**→**2**: 1,2 (C4) E2 path | -2608.53187300 | -2608.462786 |
| TS **1**→**2**: 1,2 (C4) E2 path | -2608.52430242 | -2608.460666 |
| post-reactive complex **1**→**2**: 1,2 (C4) E2 path | -2608.60195478 | -2608.537582 |
| pre-reactive complex **1**→**2**: 1,2 (C4) E1cB path | -2608.53187287 | -2608.462798 |
| TS **1**→**2**: 1,2 (C4) E1cB path | -2608.52421001 | -2608.460839 |
| Reaction intermediate **1**→**2**: 1,2 (C4) E1cB path | -2608.52977836 | -2608.465690 |
| Second TS **1**→**2**: 1,2 (C4) E1cB path | -2608.52975746 | -2608.463221 |
| post-reactive complex **1**→**2**: 1,2 (C4) E1cB path | -2608.59534017 | -2608.531731 |
| *trans*-1,4,5,6-tetrachlorocyclohexa-1,3-diene (**2**) | -2071.77870592 | -2071.729451 |
| pre-reactive complex **1**→**3**: 1,2 (C4) | - | - |
| TS **1**→**3** 1,2 (C4) | –^a^ | –^a^ |
| post-reactive complex **1**→**3**: 1,2 (C4) | - | - |
| *trans*-1,3,4,6-tetrachlorocyclohexa-1,4-diene (**3**) | -2071.77515425 | -2071.726214 |
| pre-reactive complex **1**→**4**: 1,2 (C5) | - | - |
| TS **1**→**4**: 1,2 (C5) | –^a^ | –^a^ |
| post-reactive complex **1**→**4**: 1,2 (C5) | - | - |
| *trans*-1,3,5,6-tetrachlorocyclohexa-1,4-diene (**4**) | -2071.77489377 | -2071.726683 |
| pre-reactive complex **1**→**5**: 1,2 (C5) | -2608.54064488 | -2608.473908 |
| TS **1**→**5**: 1,2 (C5) | -2608.52518400 | -2608.461202 |
| post-reactive complex **1**→**5**: 1,2 (C5) | -2608.59549036 | -2608.530268 |
| pre-reactive complex **1**→**5**: 1,2 (C3) | -2608.54064488 | -2608.473908 |
| TS **1**→**5**: 1,2 (C3) | -2608.53602484 | -2608.470253 |
| post-reactive complex **1**→**5**: 1,2 (C3) | -2608.54064488 | -2608.473908 |
| pre-reactive complex **1**→**5**: 1,4 (C3) | -2608.54064488 | -2608.473908 |
| TS **1**→**5**: 1,4 (C3) | -2608.53729252 | -2608.472314 |
| post-reactive complex **1**→**5**: 1,4 (C3) | -2608.58846835 | -2608.524990 |
| *trans*-1,3,5,6-tetrachlorocyclohexa-1,3-diene (**5**) | -2071.77772237 | -2071.728679 |
| pre-reactive complex **1**→**6**: 1,4 (C6) | -2608.54064488 | -2608.473908 |
| TS **1**→**6**: 1,4 (C6) | -2608.53718105 | -2608.472289 |
| post-reactive complex **1**→**6**: 1,4 (C6) | -2608.58714469 | -2608.523935 |
| *trans*-1,2,5,6-tetrachlorocyclohexa-1,3-diene (**6**) | -2071.76803222 | -2071.719636 |
| pre-reactive complex **1**→**7**: 1,4 (C6) | - | - |
| TS **1**→**7**: 1,4 (C6) | –^a^ | –^a^ |
| post-reactive complex **1**→**7**: 1,4 (C6) | - | - |
| *trans*-1,2,5,6-tetrachlorocyclohexa-1,3-diene (**7**) | -2071.77379002 | -2071.724986 |
| pre-reactive complex **2**→1,2,4-TCB | -2147.70731662 | -2147.652957 |
| TS **2**→1,2,4-TCB | -2147.70573048 | -2147.653882 |
| post-reactive complex **2**→1,2,4-TCB | -2147.82167425 | -2147.768512 |
| pre-reactive complex **3**→1,2,4-TCB | - | - |
| TS **3**→1,2,4-TCB | –^a^ | –^a^ |
| post-reactive complex **3**→1,2,4-TCB | - | - |
| pre-reactive complex **4**→1,2,3-TCB | -2147.70517924 | -2147.651980 |
| TS **4**→1,2,3-TCB | -2147.70516515 | -2147.651589 |
| post-reactive complex **4**→1,2,3-TCB | -2147.81203912 | -2147.762244 |
| pre-reactive complex **4**→1,3,5-TCB | - | - |
| TS **4**→1,3,5-TCB | –^a^ | –^a^ |
| post-reactive complex **4**→1,3,5-TCB | - | - |
| pre-reactive complex **5**→1,2,4-TCB | -2147.70607241 | -2147.652084 |
| TS **5**→1,2,4-TCB | -2147.70436932 | -2147.652569 |
| post-reactive complex **5**→1,2,4-TCB | -2147.82119167 | -2147.768363 |
| pre-reactive complex **5**→1,3,5-TCB | -2147.70607241 | -2147.652084 |
| TS **5**→1,3,5-TCB | -2147.70178331 | -2147.650669 |
| post-reactive complex **5**→1,3,5-TCB | -2147.82368645 | -2147.770839 |
| pre-reactive complex **6**→1,2,3-TCB | -2147.69696908 | -2147.643642 |
| TS **6**→1,2,3-TCB | -2147.69684443 | -2147.644863 |
| post-reactive complex **6**→1,2,3-TCB | -2147.82134279 | -2147.765795 |
| pre-reactive complex **6**→1,2,4-TCB | -2147.70382443 | -2147.648214 |
| TS **6**→1,2,4-TCB | -2147.69964570 | -2147.648772 |
| post-reactive complex **6**→1,2,4-TCB | -2147.82134279 | -2147.770930 |
| pre-reactive complex **7**→1,2,4-TCB | -2147.70103386 | -2147.646077 |
| TS **7**→1,2,4-TCB | -2147.69734417 | -2147.646456 |
| post-reactive complex **7**→1,2,4-TCB | -2147.82109293 | -2147.769585 |
| 1,2,3-TCB | -1610.99581172 | -1610.957765 |
| 1,2,4-TCB | -1610.99843290 | -1610.960407 |
| 1,3,5-TCB | -1611.00016806 | -1610.962423 |
| 1,3-dichlorocyclohex-2-en-1-yl localized anion | -1153.27075300 | -1153.190449 |
| TS 1,3-dichlorocyclohex-2-en-1-yl delocalized anion | -1153.26445648 | -1153.184823 |
| 1,2-dichlorocyclohex-2-en-1-yl anion | -1153.27381173 | -1153.193665 |

^a^ Structure not located.

**Table S5.** Cartesian coordinates of both pre-reactive complex and TS involved in the first stage of β-HCH elimination as calculated at different theoretical levels and medium models.

pre-reactive complex, gas-phase/HF/6-311++G(d,p)

C 1.3991948019 -0.3548567970 -0.3480973542

C 0.3815270484 -1.4001067782 0.1138104224

H 1.5065610493 -0.3883779895 -1.4230720911

Cl 0.8381897076 -3.0008790658 -0.6082328615

Cl 3.0260238060 -0.7641232868 0.3008313497

H 0.3945777800 -1.4737110828 1.1917252666

O -0.0395432175 0.0225817544 2.5329389725

H -0.0431213630 0.0439280234 3.4723100785

C -1.4318436483 0.3666719655 0.0843301286

H -1.5222644943 0.3951329130 1.1607037429

C -0.4059334228 1.4037217948 -0.3777919099

H -0.4242606772 1.4926047685 -1.4548215441

C 1.0052950594 1.0543539181 0.0995262935

H 1.0557977619 1.1244643293 1.1764289753

C -1.0261380617 -1.0386834946 -0.3650249936

H -1.0861766089 -1.1183402490 -1.4412798743

Cl -2.2073729043 -2.2409106360 0.2629600333

Cl -3.0320053525 0.7694026106 -0.6701155986

Cl -0.8681746207 3.0296861390 0.2370121623

Cl 2.1680793565 2.2351521633 -0.6394301986

pre-reactive complex, IEFPCM/HF/6-311++G(d,p)

C 1.2743432532 0.7101100617 -0.4147180153

C 1.2521816627 -0.7462252497 0.0536696520

H 1.3777888912 0.7648918955 -1.4871556711

Cl 2.6905966269 -1.6036039248 -0.6159672805

Cl 2.7310240889 1.5263285682 0.2605726140

H 1.3131839967 -0.7811353496 1.1290436017

O -0.0226649291 0.0258032998 2.7860441207

H 0.0066501203 -0.0203125354 3.7268185192

C -1.2705133350 -0.7121092276 0.0501110570

H -1.3362107807 -0.7474074818 1.1253370852

C -1.2520128079 0.7451405172 -0.4158619302

H -1.3535714724 0.8054340924 -1.4881815088

C 0.0210022238 1.4549340104 0.0496345790

H 0.0214989497 1.5280978305 1.1248744572

C -0.0184036331 -1.4597125005 -0.4131743133

H -0.0179633523 -1.5780131315 -1.4855949921

Cl -0.0424348887 -3.1282253430 0.2647602277

Cl -2.7294970601 -1.5290755314 -0.6261248461

Cl -2.6862538329 1.5997910247 0.2601432928

Cl 0.0441442790 3.1272959750 -0.6252536490

pre-reactive complex, CPCM/HF/6-311++G(d,p)

C 1.2022017293 -0.8337589517 -0.3992533633

C -0.1236634405 -1.4530788812 0.0476316520

H 1.3167917324 -0.9038364820 -1.4696209370

Cl -0.2531341512 -3.1211065171 -0.6258605547

Cl 2.5632657502 -1.7838588136 0.3007763590

H -0.1440657311 -1.5245623092 1.1227789522

O -0.0411895512 0.0091044722 2.7900342453

H -0.0282456470 -0.0333914443 3.7313357151

C -1.1997982349 0.8285098069 0.0321121530

H -1.2756840480 0.8703226977 1.1063463609

C 0.1240896715 1.4532173698 -0.4135452062

H 0.1489759014 1.5760052384 -1.4850568977

C 1.3139961127 0.6195565869 0.0669469682

H 1.3649508221 0.6501683627 1.1430152550

C -1.3163348459 -0.6243471878 -0.4343853460

H -1.4110279961 -0.6766190764 -1.5077804671

Cl -2.8342907417 -1.3356020306 0.2251763594

Cl -2.5647715585 1.7821117937 -0.6605245334

Cl 0.2521800513 3.1148910670 0.2703828484

Cl 2.8363751755 1.3330072986 -0.5849855628

pre-reactive complex, gas-phase/BLYP/6-311++G(d,p)

C 1.4154027386 -0.3595985079 -0.3734742992

C 0.3997070502 -1.4138961007 0.1169025088

H 1.5074574302 -0.3882281859 -1.4663078729

Cl 0.8570401741 -3.0704832511 -0.6231862773

Cl 3.1084568235 -0.7870278104 0.2354328939

H 0.4256004397 -1.4795434666 1.2194616884

O 0.1388294038 -0.0200343207 2.5190314656

H -0.3955703378 0.1357850362 3.3176167251

C -1.4225376659 0.3650897507 0.1087730956

H -1.5019355990 0.3904480375 1.2062601724

C -0.3969161092 1.4150854478 -0.3595188907

H -0.4366673524 1.5394113350 -1.4482215872

C 1.0273115615 1.0560811864 0.1056592647

H 1.0821681024 1.1095608140 1.2083715074

C -1.0241752789 -1.0518454569 -0.3474743455

H -1.1212761026 -1.1518306977 -1.4350347613

Cl -2.2346079518 -2.2832640836 0.3311758320

Cl -3.0897612195 0.7844600034 -0.6673143607

Cl -0.8702936384 3.0815498620 0.3046228963

Cl 2.2201795314 2.2859914085 -0.6480646553

pre-reactive complex, IEFPCM/BLYP/6-311++G(d,p)

C -1.407772476649 -0.381796146560 -0.427700919175

C -1.035127379041 1.033857190882 0.060705175046

H -1.523434735094 -0.411598541398 -1.516257292614

Cl -2.256665830869 2.256953114482 -0.614983700454

Cl -3.078835032520 -0.835667218943 0.234826922773

H -1.080672070264 1.069803047887 1.159052317614

O -0.180947637576 -0.057425185281 2.712169524056

H 0.523638809380 0.067416332717 3.374219233442

C 1.422921083752 0.387306215529 0.057918939717

H 1.494740110707 0.405581044101 1.152490284554

C 1.050584205926 -1.034675045217 -0.409319515111

H 1.156356023896 -1.135948564047 -1.494106001466

C -0.369114054161 -1.414518187497 0.058213321335

H -0.389068857341 -1.466436573085 1.157885367624

C 0.381335891668 1.425955056338 -0.406657452858

H 0.419640497214 1.567679615765 -1.491385109572

Cl 0.819804248959 3.083230850472 0.301439929867

Cl 3.085444153029 0.840203675649 -0.636610945730

Cl 2.268636191241 -2.242004567432 0.297908685398

Cl -0.803613142259 -3.087204114361 -0.620109764446

pre-reactive complex, CPCM/BLYP/6-311++G(d,p)

C 1.044148934950 -1.010344870029 -0.436341719177

C -0.360044657655 -1.414712890315 0.058945369291

H 1.124347907832 -1.088520644473 -1.525812343273

Cl -0.790380412519 -3.091334244864 -0.612896325601

Cl 2.294299130230 -2.215916025675 0.212148761249

H -0.362484300008 -1.468147101600 1.157959556524

O 0.180412035642 -0.171245990321 2.718094831556

H -0.277438430882 0.376221754184 3.382147733497

C -1.064194544420 1.026934889749 0.058430320904

H -1.123095214384 1.083743510293 1.151612475750

C 0.349182179826 1.435286192171 -0.405187415806

H 0.382440613123 1.583657518536 -1.489658104453

C 1.401572637293 0.406901215790 0.056763416649

H 1.455937440061 0.408541816567 1.157081110972

C -1.424934102853 -0.399575695878 -0.404645144354

H -1.572229566734 -0.439540501546 -1.488623367823

Cl -3.070548505512 -0.869250256312 0.311618412810

Cl -2.301706728376 2.223653657081 -0.641222325808

Cl 0.763368691040 3.094920307549 0.311883714455

Cl 3.060975893345 0.896172359094 -0.619434957362

pre-reactive complex, gas-phase/B3LYP/6-311++G(d,p)

C 1.3992211795 -0.3547714724 -0.3573688619

C 0.3805867362 -1.3967088172 0.1172880502

H 1.5103810096 -0.3884491940 -1.4423255515

Cl 0.8466238943 -3.0317871513 -0.5710132270

Cl 3.0533396801 -0.7716911452 0.2870622735

H 0.3820838038 -1.4296187887 1.2173266069

O -0.0377085176 0.0205226506 2.4821345918

H -0.0399331254 0.0440700844 3.4429415929

C -1.4289511432 0.3662009034 0.0884433548

H -1.4781337433 0.3842066449 1.1877736955

C -0.4061481911 1.4034819053 -0.3869595526

H -0.4250371866 1.4957296658 -1.4740090747

C 1.0025877312 1.0517056307 0.1040904239

H 1.0220087575 1.0902599755 1.2038110542

C -1.0263608918 -1.0382884803 -0.3741068270

H -1.0892458965 -1.1207238733 -1.4603139503

Cl -2.2266127717 -2.2609979638 0.2502616444

Cl -3.0646472300 0.7781647369 -0.6325820855

Cl -0.8759673845 3.0566913442 0.2226741515

Cl 2.1903252896 2.2597143445 -0.6004173091

pre-reactive complex, IEFPCM/B3LYP/6-311++G(d,p)

C -1.411631988089 -0.363157638910 -0.417308140806

C -1.016566994476 1.041424209099 0.052229358701

H -1.533121638059 -0.397329666577 -1.498995297678

Cl -2.209738058941 2.255762703793 -0.605148119035

Cl -3.053067307739 -0.783404248249 0.260696329896

H -1.048046864631 1.077303036635 1.144647294689

O 0.016382527476 0.018783337836 2.666594595081

H -0.045198016380 -0.016992406325 3.627531387594

C 1.410777091403 0.364237575002 0.054067614842

H 1.452406997790 0.375303682597 1.146539755475

C 1.023199329024 -1.040211529240 -0.422927959931

H 1.104329816182 -1.125543732759 -1.505756234520

C -0.387216300696 -1.398802563158 0.058636785794

H -0.394255284632 -1.436202920071 1.151560702196

C 0.393675209409 1.405812404817 -0.425228515338

H 0.426209586325 1.517404766828 -1.508172929715

Cl 0.849328949955 3.044132341737 0.236561506132

Cl 3.062204928023 0.790491303164 -0.594278865760

Cl 2.212688758897 -2.253280666087 0.242780653079

Cl -0.848246740882 -3.043048990074 -0.584804920695

pre-reactive complex, CPCM/B3LYP/6-311++G(d,p)

C 1.4118083413 -0.3692089531 -0.4161810943

C 0.3839335608 -1.4036149319 0.0545862013

H 1.5331579864 -0.3979180180 -1.4981605968

Cl 0.8366110413 -3.0464784519 -0.5971006787

Cl 3.0517946144 -0.8002269284 0.2587520081

H 0.3922733325 -1.4482119016 1.1473103611

O -0.0007254402 -0.0148719101 2.6684177412

H -0.0413197213 -0.0113001032 3.6313629093

C -1.4086339617 0.3676435937 0.0515387474

H -1.4536013342 0.3753855731 1.1443705973

C -0.3863031218 1.4083691702 -0.4175260991

H -0.4153327404 1.5308581692 -1.4994943000

C 1.0204390111 1.0337048157 0.0626379469

H 1.0459974718 1.0608101456 1.1558185701

C -1.0247343017 -1.0375092161 -0.4255975702

H -1.1064077435 -1.1227115973 -1.5082374696

Cl -2.2204553680 -2.2456428478 0.2394453723

Cl -3.0555414405 0.8009583021 -0.6036611974

Cl -0.8378057682 3.0423166866 0.2591528586

Cl 2.2219415818 2.2486384032 -0.5772953075

pre-reactive complex, gas-phase/M06-2X/6-311++G(d,p)

C 1.3924140648 -0.3519643450 -0.3510459774

C 0.3808439614 -1.3937520825 0.1219964919

H 1.4907012149 -0.3826574218 -1.4392536703

Cl 0.8368152532 -2.9992163072 -0.5758661741

Cl 3.0214993027 -0.7625982663 0.2903725782

H 0.3853865775 -1.4314610920 1.2201595210

O -0.0370819617 0.0253062833 2.4547770314

H -0.0448616929 0.0303773432 3.4128895485

C -1.4266612670 0.3661398585 0.0934982895

H -1.4826936578 0.3872301682 1.1906422464

C -0.4041268825 1.3969012541 -0.3804707164

H -0.4195114985 1.4749273955 -1.4707725566

C 1.0010278587 1.0510625251 0.1082534668

H 1.0249700849 1.0937951662 1.2060118442

C -1.0210043367 -1.0332012553 -0.3652627395

H -1.0770823079 -1.1076512835 -1.4544436024

Cl -2.2029070451 -2.2367011125 0.2598911640

Cl -3.0312954026 0.7697323400 -0.6373496818

Cl -0.8659642849 3.0259111951 0.2249786669

Cl 2.1679440193 2.2355306371 -0.6042947305

pre-reactive complex, IEFPCM/M06-2X/6-311++G(d,p)

C -1.4047330284 -0.3611524332 -0.4169960319

C -1.0147123528 1.0355716735 0.0603867757

H -1.5049787386 -0.3894373080 -1.5035600335

Cl -2.1900486832 2.2343279109 -0.5915941204

Cl -3.0271082373 -0.7759451876 0.2429381682

H -1.0411919488 1.0622220091 1.1540286398

O -0.0095435752 0.0000618729 2.6142712416

H 0.0125484801 0.0347429863 3.5743600506

C 1.4037462246 0.3610679886 0.0629008615

H 1.4390459157 0.3721888611 1.1561367589

C 1.0157575433 -1.0372252328 -0.4118043958

H 1.0922936503 -1.1165769640 -1.4977953568

C -0.3898064374 -1.3950977474 0.0655000692

H -0.4009884824 -1.4284867560 1.1589816662

C 0.3899452967 1.3970665912 -0.4173454395

H 0.4183178509 1.4980437527 -1.5037292324

Cl 0.8399743399 3.0101940148 0.2433282341

Cl 3.0304320485 0.7769611552 -0.5874399844

Cl 2.1851113101 -2.2307930561 0.2572222250

Cl -0.8399471760 -3.0150531314 -0.5805651159

pre-reactive complex, CPCM/M06-2X/6-311++G(d,p)

C -1.039401598716 -1.014849444074 -0.414248610878

C -1.396009677224 0.391193521790 0.062451724104

H -1.117566588458 -1.091879127218 -1.500275588832

Cl -3.013510367560 0.844469859102 -0.586865880881

Cl -2.236151408789 -2.182655089297 0.253695629816

H -1.431760242490 0.401684294117 1.155818505032

O 0.009012200307 0.015881332023 2.614774195200

H 0.006488943556 -0.008004825317 3.575403801975

C 1.036453484291 1.011044264402 0.061840007199

H 1.060579556029 1.033181633398 1.155697526554

C 1.396440993851 -0.393053194071 -0.417936689328

H 1.498255651623 -0.422565436661 -1.504365595921

C 0.357912574256 -1.404136894631 0.063263850535

H 0.367682993515 -1.435137905196 1.156901875125

C -0.359177092014 1.405168255791 -0.416271809388

H -0.385702444282 1.508957668342 -1.502491934774

Cl -0.773206297097 3.026442155768 0.248569332457

Cl 2.238791962177 2.185554460785 -0.584544999357

Cl 3.008396186154 -0.844875628370 0.245012409309

Cl 0.773068170871 -3.033853900685 -0.581178747946

TS, gas-phase/HF/6-311++G(d,p)

C 0.7855498850 -1.1836721734 -0.3820086866

C -0.6480072884 -1.2130149364 0.1394686851

H 0.8550197430 -1.2833412379 -1.4589055298

Cl -1.4270242362 -2.7494044095 -0.4688864177

Cl 1.7209557186 -2.5906189284 0.2618284800

H -0.5927233541 -1.0236719644 1.4533875586

O -0.3373007891 -0.4648131032 2.5539525642

H -0.6173403421 -0.9563784576 3.3047818253

C -0.7835188289 1.2779230496 0.0042414159

H -0.9111705451 1.3863543972 1.0681855559

C 0.6980952880 1.3427583177 -0.3718380074

H 0.7983679445 1.4681676484 -1.4392171962

C 1.4743941794 0.1036119645 0.0783824470

H 1.5814395683 0.0901340557 1.1500067649

C -1.4181678986 -0.0374899450 -0.4545611779

H -1.4836463966 -0.0668009868 -1.5359136944

Cl -3.1383030207 -0.0633192462 0.1017779268

Cl -1.6218407570 2.6756990619 -0.7871537066

Cl 1.4402836114 2.8156696128 0.3553607561

Cl 3.1388285185 0.1995752810 -0.6310965634

TS, IEFPCM/HF/6-311++G(d,p)

C -0.6520980226 -1.2071132161 -0.3318978651

C -1.3144148788 0.0712978383 0.168276087

H -0.7465042173 -1.3556093272 -1.3985375168

Cl -3.0246032825 0.0828555839 -0.5227486413

Cl -1.4796217635 -2.6482200249 0.4015876462

H -1.3977726203 0.1082268862 1.5354279143

O -1.4209770561 0.1618093260 2.7598638379

H -1.6110937456 -0.6981607784 3.0957063281

C 0.8742197971 1.2889956935 -0.0011445189

H 0.9588002190 1.4420994262 1.0620216683

C 1.5754506828 -0.0075090180 -0.4119311133

H 1.6916335373 -0.0420888464 -1.4835172543

C 0.8219371638 -1.2465015244 0.0771201441

H 0.9054854724 -1.3381538521 1.1474949145

C -0.5987487752 1.2837129853 -0.4156321889

H -0.6817986548 1.3585467795 -1.4910210111

Cl -1.3681957003 2.8054428234 0.2061258844

Cl 1.7289422503 2.6747814234 -0.7772685578

Cl 3.2438081579 -0.0218642994 0.2670498098

Cl 1.6137484362 -2.7118828787 -0.6148255672

TS, CPCM/HF/6-311++G(d,p)

C -0.6032375499 -1.2820673644 -0.4145551781

C -1.3149326281 -0.0664139875 0.1674723971

H -0.6866193179 -1.3583211293 -1.4897622222

Cl -3.0249848429 -0.0734003767 -0.5242448841

Cl -1.3777952166 -2.8005962943 0.2095308188

H -1.3991800844 -0.1002594238 1.5350787303

O -1.4283216878 -0.1590020243 2.7584601965

H -1.5980260044 0.7033173908 3.0992037921

C 0.8256376537 1.2439442802 0.0760334827

H 0.9085615424 1.3361740486 1.1464122643

C 1.5754698438 0.0020669190 -0.4114644097

H 1.6929025460 0.0355989580 -1.4829112847

C 0.8697767748 -1.2919443096 -0.0002870795

H 0.9542673721 -1.4451670397 1.0628709835

C -0.6482230930 1.2091851550 -0.3340576697

H -0.7416543636 1.3572643863 -1.4008003794

Cl -1.4713203858 2.653722040 7 0.3981380426

Cl 1.6229045420 2.7061930002 -0.6161508154

Cl 3.2432787567 0.0113050072 0.2692366701

Cl 1.7196931429 -2.6809342363 -0.7760534550

TS, gas-phase/BLYP/6-311++G(d,p)

C 0.8520672203 -1.1599248942 -0.4024636573

C -0.5950690638 -1.2791074457 0.0687027963

H 0.9812071700 -1.2576997061 -1.4891089462

Cl -1.3181176468 -2.8402275529 -0.6832445897

Cl 1.8700267031 -2.5950416984 0.2555697987

H -0.5544744828 -1.1739170903 1.3777158114

O -0.2649954060 -0.6184265102 2.5534354756

H -1.1038940157 -0.5766326916 3.0460544651

C -0.8322860899 1.2374305774 0.0428741658

H -0.9473314938 1.3304347494 1.1273470268

C 0.6519381976 1.3729277735 -0.3492835555

H 0.7530382986 1.5192091169 -1.4306083731

C 1.4792672777 0.1554031431 0.1087082153

H 1.5638975643 0.1287672636 1.1994977574

C -1.4283949561 -0.1057807668 -0.4247331445

H -1.6160548698 -0.1028642239 -1.5061983748

Cl -3.1836716458 -0.2236129775 0.2798353107

Cl -1.7778657555 2.6459102891 -0.7332266966

Cl 1.3492987638 2.9253014084 0.3937736224

Cl 3.2050022307 0.3364872361 -0.5643441079

TS, IEFPCM/BLYP/6-311++G(d,p)

C 0.2596310794 -1.3900726048 -0.3770645700

C -1.0528517983 -0.8009255029 0.1048429753

H 0.2876525281 -1.6210820301 -1.4481339931

Cl -2.4409548093 -1.8148146671 -0.6665822102

Cl 0.5095140108 -3.1105749344 0.3955117349

H -1.1657248160 -0.8831118501 1.4557359242

O -1.2062919469 -0.9168096891 2.7358847476

H -1.8083752143 -0.1865468662 2.9676217394

C -0.0470339467 1.5296583488 0.0448623947

H -0.0214188325 1.6811038222 1.1279843617

C 1.2893320411 0.9393261890 -0.4512296405

H 1.3600904353 1.0020249338 -1.5419606233

C 1.4676745275 -0.5183655777 0.0229789023

H 1.6276269632 -0.5556076263 1.1044068031

C -1.2394915578 0.6346704333 -0.3480661754

H -1.4793465321 0.7496894124 -1.4110651991

Cl -2.7961419483 1.3634688899 0.4791374435

Cl -0.2669173505 3.2193316043 -0.6794407394

Cl 2.6899361297 1.9705086321 0.1846673205

Cl 3.0146030375 -1.2083639169 -0.7241421963

TS, CPCM/BLYP/6-311++G(d,p)

C 0.2613908968 -1.3900487442 -0.3778122819

C -1.0527033724 -0.8039520019 0.1027265479

H 0.2912989531 -1.6216795472 -1.4486610972

Cl -2.4381318118 -1.8215877933 -0.6689600152

Cl 0.5143379071 -3.1100712262 0.3960598018

H -1.1637912329 -0.8850172456 1.4550426462

O -1.1991584294 -0.9189839699 2.7337485242

H -1.7848454386 -0.1769177283 2.9700224992

C -0.0525587458 1.5286679940 0.0445007437

H -0.0286951758 1.6795910425 1.1277417470

C 1.2859414628 0.9417294047 -0.4499190543

H 1.3585270797 1.0052841704 -1.5404317483

C 1.4669252614 -0.5157019964 0.0239865721

H 1.6251858198 -0.5529269674 1.1056916985

C -1.2424967401 0.6310661940 -0.3501205271

H -1.4820880422 0.7461260516 -1.4131369521

Cl -2.8017389831 1.3559219612 0.4766630588

Cl -0.2755142464 3.2181866921 -0.6791954418

Cl 2.6831483359 1.9759032946 0.1890259296

Cl 3.0164785018 -1.2020825848 -0.7210236511

TS, gas-phase/B3LYP/6-311++G(d,p)

C 0.7916867731 -1.1807997037 -0.3735407905

C -0.6468908092 -1.2314244926 0.1154931039

H 0.8992817862 -1.2969967419 -1.4562803475

Cl -1.4089036011 -2.7841421323 -0.5175486087

Cl 1.7475702013 -2.6012455638 0.3028797973

H -0.5903572897 -1.0245415204 1.4286283502

O -0.3053996112 -0.3828424220 2.5128831055

H -0.5833073564 -0.8881561700 3.2823348515

C -0.8071307696 1.2623515145 0.0188748239

H -0.9304076029 1.3479698057 1.0981197517

C 0.6738547143 1.3456094442 -0.3639792155

H 0.7755968539 1.4690917316 -1.4422457484

C 1.4599616065 0.1159215619 0.1015704012

H 1.5392093217 0.0994236127 1.1881975837

C -1.4277212156 -0.0583213095 -0.4549708341

H -1.5212372539 -0.0723820659 -1.5451412457

Cl -3.1750967641 -0.1119465143 0.1216871945

Cl -1.6810238529 2.6760360000 -0.752834115

Cl 1.4113298720 2.8554483990 0.3518576112

Cl 3.1585129974 0.2284565668 -0.5767376693

TS, IEFPCM/B3LYP/6-311++G(d,p)

C 0.6159263419 1.2844834552 -0.4085256011

C 1.3193046564 0.0634776246 0.1482842303

H 0.7209873708 1.4081219077 -1.4881054230

Cl 3.0383871030 0.0539442351 -0.5524804872

Cl 1.4022954060 2.8193330979 0.2683054811

H 1.3973989929 0.1003927126 1.5192284722

O 1.3990994550 0.1475215195 2.7640051532

H 1.4929573162 -0.7665677587 3.0553017990

C -0.8378213798 -1.2318462623 0.0743520001

H -0.9431712129 -1.3078747606 1.1546162480

C -1.5692564252 0.0091836626 -0.4475616298

H -1.6566801080 -0.0286826080 -1.5323832025

C -0.8652769222 1.2995820237 -0.0150154416

H -0.9678669842 1.4487452945 1.0577381068

C 0.6450223417 -1.2131861730 -0.3115886193

H 0.7629726129 -1.4245185525 -1.3758855096

Cl 1.4595815275 -2.6701482409 0.4983767872

Cl -1.6395310006 -2.7224687731 -0.6081522346

Cl -3.2815649682 0.0110925062 0.1804692860

Cl -1.7043041230 2.7194690896 -0.7960664151

TS, CPCM/B3LYP/6-311++G(d,p)

C 0.6150153693 1.2865541853 -0.4078277500

C 1.3206047957 0.0660394345 0.1467768523

H 0.7206835963 1.4130584783 -1.4869444543

Cl 3.0391041301 0.0598673970 -0.5560618038

Cl 1.3981331121 2.8221425449 0.2731301353

H 1.3992671276 0.1006441864 1.5186333113

O 1.4018107778 0.1448221316 2.7624627772

H 1.4713877207 -0.7720592759 3.0517663783

C -0.8344529799 -1.2325052128 0.0750397215

H -0.9386098075 -1.3080313616 1.1554691003

C -1.5684064625 0.0070678815 -0.4467886707

H -1.6568122481 -0.0313779620 -1.5314788394

C -0.8663833955 1.2988425774 -0.0150040090

H -0.9696538489 1.4485824536 1.0575942092

C 0.6479268183 -1.2115546294 -0.3126110250

H 0.7652517131 -1.4231943701 -1.3768682642

Cl 1.4656923727 -2.6673626572 0.4970241138

Cl -1.6344091684 -2.7249651789 -0.6054346029

Cl -3.2801805801 0.0064238475 0.1829628224

Cl -1.7075090428 2.7170595299 -0.7969270022

TS, gas-phase/M06-2X/6-311++G(d,p)

C -0.702867315552 -1.235641728086 -0.422993174512

C -1.409629787949 0.009765127753 0.085634081183

H -0.714699773866 -1.329340564745 -1.514715540860

Cl -3.090849517792 0.019735014354 -0.586875458515

Cl -1.555912305511 -2.714539773087 0.173473753810

H -1.229919190323 0.009576954171 1.369581777344

O -0.480369910254 0.010045232341 2.461220060143

H -0.989987909488 -0.048183007396 3.271087049991

C 0.749584071388 1.258851506136 0.102125124738

H 0.732281240864 1.345150207044 1.190516099764

C 1.488163822753 -0.008750422129 -0.320550908358

H 1.642154288598 -0.008287581805 -1.401896396595

C 0.733906105552 -1.267888751685 0.099491466045

H 0.717473251743 -1.355019159593 1.187673779350

C -0.686034000233 1.244910311073 -0.424749139658

H -0.692358521258 1.334739210792 -1.516909990675

Cl -1.522312031502 2.736136669741 0.162887549804

Cl 1.632247842555 2.683782859574 -0.573159515036

Cl 3.135853769922 -0.020285618894 0.405370193904

Cl 1.596996870346 -2.702678485504 -0.580207811868

TS, IEFPCM/M06-2X/6-311++G(d,p)

C 0.2439725576 1.3838474730 -0.4301039122

C 1.2621406463 0.3326695125 -0.2656453331

H 0.1074677180 1.7627705221 -1.4469578380

Cl 2.6881407295 0.7153008763 -1.3207717417

Cl 0.7950831415 2.9706122364 0.4417669896

H 1.8941564378 0.4021323879 1.5181231072

O 2.2271873658 0.3474997799 2.4562298653

H 2.5007188706 -0.5711204891 2.5364997311

C -0.5028400528 -1.3990951957 0.1123762518

H -0.4309262485 -1.4478746788 1.1990193145

C -1.5500579867 -0.3661098999 -0.2922261549

H -1.7086117797 -0.3747623987 -1.3715270153

C -1.1050436038 1.0146706636 0.1800232142

H -1.0495839000 1.0347160044 1.2684815183

C 0.8533648006 -1.0583118030 -0.4967794363

H 0.9124293682 -1.4172112356 -1.5276374303

Cl 2.0966820482 -2.2589775122 0.3174557599

Cl -1.0161011667 -3.0367659695 -0.4291686393

Cl -3.1311535139 -0.7816806091 0.4609454310

Cl -2.3342204319 2.2446773355 -0.2822226820

TS, CPCM/M06-2X/6-311++G(d,p)

C 0.2441035883 1.3842634629 -0.4307003214

C 1.2624572293 0.3334034238 -0.2687033935

H 0.1072188274 1.7647872764 -1.4468158293

Cl 2.6864165750 0.7162654755 -1.3264614793

Cl 0.7957167557 2.9713191618 0.4426236650

H 1.8937045545 0.4007551550 1.5181890191

O 2.2253567332 0.3454330260 2.4564405438

H 2.4912728981 -0.5752050090 2.5392800502

C -0.5013404560 -1.3985090705 0.1133357401

H -0.4285128541 -1.4459232613 1.1999866695

C -1.5492541968 -0.3662999048 -0.2917019464

H -1.7080063560 -0.3756661187 -1.3709469986

C -1.1045655580 1.0148003496 0.1799001888

H -1.0489508305 1.0351615877 1.2683378852

C 0.8540558796 -1.0577370373 -0.4975976639

H 0.9124082956 -1.4179644060 -1.5279700199

Cl 2.0991300476 -2.2579697360 0.3165730449

Cl -1.0145449024 -3.0369915844 -0.4256828415

Cl -3.1300073800 -0.7816915669 0.4620556817

Cl -2.3338538504 2.2447557762 -0.2822609945

**Table S6.** Cartesian coordinates of all structures at CPCM(water)/M06-2X/6-311++G(d,p) level.

β-HCH (all-equatorial conformer)

C 0.000000 1.455793 0.2334200

C -1.260754 -0.727897 0.2334200

C 1.260754 -0.727897 0.2334200

C 0.000000 -1.455793 -0.2334200

C 1.260754 0.727897 -0.2334200

C -1.260754 0.727897 -0.2334200

H 0.000000 -1.551579 -1.3201300

H 1.343707 0.775790 -1.3201300

H -1.343707 0.775790 -1.3201300

H 0.000000 1.551579 1.3201300

H -1.343707 -0.775790 1.3201300

H 1.343707 -0.775790 1.3201300

Cl 0.000000 3.123161 -0.4266540

Cl 2.704737 -1.561581 -0.4266540

Cl -2.704737 -1.561581 -0.4266540

Cl 0.000000 -3.123161 0.4266540

Cl -2.704737 1.561581 0.4266540

Cl 2.704737 1.561581 0.4266540

β-HCH (all-axial conformer)

C 0.157403 1.064736 -1.0574670

C -0.155593 -0.383859 -1.4504750

C 0.157871 -1.448413 -0.3929020

C -0.157404 -1.064737 1.0574660

C 0.155592 0.383858 1.4504750

C -0.157870 1.448413 0.3929010

H -0.382959 -2.357859 -0.6398510

H 0.384367 -0.624913 -2.3620060

H -0.382810 1.732919 -1.7224600

H 0.382809 -1.732920 1.7224590

H -0.384370 0.624912 2.3620050

H 0.382963 2.357858 0.6398490

Cl -1.897939 -1.411258 1.3973100

Cl 1.898381 -1.918698 -0.5192760

Cl -1.896617 -0.508511 -1.9186690

Cl 1.897939 1.411256 -1.3973140

Cl -1.898378 1.918702 0.5192750

Cl 1.896615 0.508510 1.9186740

hydroxide anion (HO^-^)

O 0.000000 0.000000 0.1067680

H 0.000000 0.000000 -0.8541430

water (H_2_O)

O 0.000000 0.000000 0.1177760

H 0.000000 0.759695 -0.4711050

H 0.000000 -0.759695 -0.4711050

chloride anion (Cl^-^)

Cl 0.000000 0.000000 0.0000000

pre-reactive complex β-HCH →**1**: 1,2

C -1.404625591021 -0.362252253032 -0.416307657260

C -1.013885431539 1.034859764816 0.059791763513

H -1.509031489533 -0.390541905105 -1.502449351314

Cl -2.189899894783 2.232677552150 -0.591564919840

Cl -3.023967146452 -0.777996538628 0.251212232338

H -1.041347776822 1.061641070019 1.152935267833

O -0.012973814700 -0.001630795948 2.612299712436

H 0.008580060207 0.032928786210 3.572303564510

C 1.404082061022 0.361452517892 0.061724851031

H 1.437649132712 0.370158985779 1.155138319574

C 1.016159131633 -1.036298199597 -0.414776809422

H 1.090670095597 -1.116204189407 -1.500622139632

C -0.388616348986 -1.395614944224 0.063323622828

H -0.399810170094 -1.430382432497 1.156603051309

C 0.390918911997 1.399252682232 -0.415706297461

H 0.422272988512 1.507064831475 -1.501480427292

Cl 0.838232084685 3.009445711059 0.256012162520

Cl 3.031236487964 0.778661586141 -0.586938583967

Cl 2.187553291733 -2.229411138756 0.252405359118

Cl -0.839082582178 -3.015130090564 -0.584678720821

TS β-HCH → **1**: 1,2

C 0.2441035883 1.3842634629 -0.4307003214

C 1.2624572293 0.3334034238 -0.2687033935

H 0.1072188274 1.7647872764 -1.4468158293

Cl 2.6864165750 0.7162654755 -1.3264614793

Cl 0.7957167557 2.9713191618 0.4426236650

H 1.8937045545 0.4007551550 1.5181890191

O 2.2253567332 0.3454330260 2.4564405438

H 2.4912728981 -0.5752050090 2.5392800502

C -0.5013404560 -1.3985090705 0.1133357401

H -0.4285128541 -1.4459232613 1.1999866695

C -1.5492541968 -0.3662999048 -0.2917019464

H -1.7080063560 -0.3756661187 -1.3709469986

C -1.1045655580 1.0148003496 0.1799001888

H -1.0489508305 1.0351615877 1.2683378852

C 0.8540558796 -1.0577370373 -0.4975976639

H 0.9124082956 -1.4179644060 -1.5279700199

Cl 2.0991300476 -2.2579697360 0.3165730449

Cl -1.0145449024 -3.0369915844 -0.4256828415

Cl -3.1300073800 -0.7816915669 0.4620556817

Cl -2.3338538504 2.2447557762 -0.2822609945

post-reactive complex β-HCH →**1**: 1,2

C -0.1981351243 -1.5539456349 -0.7731828480

C -1.0536744745 -0.3746172493 -1.1335528666

H 0.1624888123 -2.0419887448 -1.6797571279

Cl -2.5252735288 -0.7432197324 -1.9894926258

Cl -1.1718404516 -2.8166685153 0.0817985319

H -1.8708043409 -0.7556231575 1.8946347381

O -1.1934011406 -0.1645238405 2.2376299194

H -1.5247952782 0.7341389055 2.0513425211

C 0.5440346028 1.2668089214 -0.2032387985

H 0.3476378159 1.5325178832 0.8362610225

C 1.5920657545 0.1637170477 -0.3025677121

H 1.9805366182 0.0937520473 -1.3193927527

C 0.9689464876 -1.1586602272 0.1363305949

H 0.6157214898 -1.0674919358 1.1631280859

C -0.7304211079 0.8878317307 -0.8895004870

H -1.3959315726 1.6941088161 -1.1695976830

Cl -2.0433578273 2.8122032356 1.5972276741

Cl 1.1799008902 2.7756902392 -0.9677602463

Cl 2.9861346435 0.5731760164 0.7520093861

Cl 2.1799188521 -2.4849032254 0.1067704940

*rel*-(3*R*,4*S*,5*R*,6*S*)-1,3,4,5,6-pentachlorocyclohex-1-ene (**1**)

C -0.145741 -1.752222 0.3187690

C -1.228770 -1.073163 -0.0359760

C -1.254513 0.407786 -0.2892260

C 0.038225 1.082944 0.1786330

C 1.261633 0.252250 -0.2027190

C 1.191704 -1.102222 0.4963010

H -0.198389 -2.822035 0.4787220

H 0.012477 1.227845 1.2591930

H 1.309489 0.117935 -1.2841060

H 1.442817 -1.000420 1.5530520

H -1.428997 0.599524 -1.3488240

Cl -2.724399 -1.914037 -0.3179110

Cl 2.455792 -2.195170 -0.1870510

Cl 2.770347 1.090819 0.2803580

Cl 0.119213 2.719336 -0.5497310

Cl -2.639342 1.180517 0.5712340

pre-reactive complex **1**→**2**: 1,2 (C4) E2 path

C 0.176094 1.886169 -0.216300

C -0.994394 1.216928 -0.178338

C -1.170998 -0.200638 -0.180619

C 0.050612 -0.981268 -0.490288

C 1.316816 -0.306849 0.042355

C 1.441236 1.125928 -0.468364

H 0.233201 2.949527 -0.035489

H 0.170591 -1.239878 -1.545606

H 1.299483 -0.304747 1.132523

H 1.757035 1.135922 -1.513492

Cl -2.454118 2.190989 0.038066

Cl 2.814452 1.939388 0.408117

Cl 2.777693 -1.250968 -0.439811

Cl -0.049676 -2.671410 0.299802

Cl -2.563734 -0.812201 -1.146112

H -1.615340 -0.563950 1.660488

O -1.785381 -0.807859 2.605985

H -1.396603 -1.684192 2.681959

TS **1**→**2**: 1,2 (C4) E2 path

C 0.223375 -1.732425 -0.5586720

C 1.292590 -1.083254 -0.1172370

C 1.306985 0.385176 0.2003330

C 0.074255 1.083661 -0.3792270

C -1.169182 0.279599 -0.0564960

C -1.093011 -1.051395 -0.7651930

H 0.273547 -2.796081 -0.7575170

H 0.218758 1.269779 -1.4472610

H -1.338060 -0.998853 -1.8290710

H 1.352702 0.531224 1.2802020

Cl 2.761015 -1.961964 0.2177310

Cl -2.385464 -2.174948 -0.0991120

Cl -2.632839 1.155087 -0.6923110

Cl -0.019833 2.732731 0.3680160

Cl 2.794004 1.170912 -0.4646280

H -1.267759 0.120073 1.2567640

O -1.264358 -0.028478 2.5311580

H -1.721437 -0.857390 2.7017400

post-reactive complex: 1,2 (C4) E2 path

C -0.620539 -1.081413 -1.2040890

C 0.497269 -1.356887 -0.5385900

C 1.311316 -0.320322 0.1736920

C 0.912568 1.139820 -0.1326510

C -0.424348 1.304985 -0.7930790

C -1.094107 0.292303 -1.3373630

H -1.230403 -1.876424 -1.6114370

H 1.657322 1.594403 -0.7870120

H -2.008153 0.471027 -1.8880870

H 1.216816 -0.510572 1.2410370

Cl 0.971010 -3.004146 -0.2426960

Cl -3.650406 -1.308669 0.4161390

Cl -0.955797 2.945901 -1.0192460

Cl 1.039891 2.100553 1.3986890

Cl 3.079983 -0.509534 -0.1838980

H -1.193609 0.522669 2.2252080

O -1.043863 -0.401811 2.0068950

H -1.823613 -0.667316 1.4848420

pre-reactive complex **1**→**2**: 1,2 (C4) E1cB

C 0.176094 1.886169 -0.216300

C -0.994394 1.216928 -0.178338

C -1.170998 -0.200638 -0.180619

C 0.050612 -0.981268 -0.490288

C 1.316816 -0.306849 0.042355

C 1.441236 1.125928 -0.468364

H 0.233201 2.949527 -0.035489

H 0.170591 -1.239878 -1.545606

H 1.299483 -0.304747 1.132523

H 1.757035 1.135922 -1.513492

Cl -2.454118 2.190989 0.038066

Cl 2.814452 1.939388 0.408117

Cl 2.777693 -1.250968 -0.439811

Cl -0.049676 -2.671410 0.299802

Cl -2.563734 -0.812201 -1.146112

H -1.615340 -0.563950 1.660488

O -1.785381 -0.807859 2.605985

H -1.396603 -1.684192 2.681959

TS **1**→**2**: 1,2 (C4) E1cB path

C 0.249842 -1.764911 -0.5015010

C 1.307980 -1.078664 -0.0907770

C 1.296348 0.399675 0.1726200

C 0.040037 1.053846 -0.4083940

C -1.182921 0.230234 -0.0531830

C -1.080187 -1.116496 -0.7279170

H 0.320043 -2.834258 -0.6598060

H 0.167471 1.221765 -1.4813660

H -1.319285 -1.091714 -1.7944250

H 1.359345 0.585056 1.2457590

Cl 2.795847 -1.913653 0.2705720

Cl -2.353341 -2.247658 -0.0454080

Cl -2.666042 1.060708 -0.7049770

Cl -0.084580 2.716241 0.3083610

Cl 2.755798 1.191628 -0.5456470

H -1.252382 0.159582 1.2731870

O -1.221541 0.114610 2.5535370

H -0.900036 0.977074 2.8339340

Reaction intermediate **1**→**2**: 1,2 (C4) E1cB path

C -0.287995 1.782203 -0.5011010

C -1.334761 1.072853 -0.1014880

C -1.281666 -0.402285 0.1709080

C -0.021701 -1.031999 -0.4296080

C 1.190162 -0.198789 -0.1413040

C 1.055126 1.163988 -0.7136600

H -0.379636 2.850784 -0.6537940

H -0.195205 -1.278106 -1.4833160

H 1.364865 1.275316 -1.7569690

H -1.323714 -0.580615 1.2461810

Cl -2.844090 1.874937 0.2515320

Cl 2.281728 2.340955 0.1123460

Cl 2.629641 -0.991555 -0.9567880

Cl 0.148025 -2.696927 0.3455080

Cl -2.738765 -1.237691 -0.5060760

H 1.368155 -0.262793 1.6547450

O 1.383751 -0.371480 2.6627700

H 1.079360 -1.273799 2.7976430

Second TS **1**→**2**: 1,2 (C4) E1cB path

C -0.326390 1.780236 -0.5172810

C -1.361223 1.059328 -0.1066980

C -1.279071 -0.410229 0.1907260

C -0.025795 -1.033538 -0.4292710

C 1.182141 -0.184944 -0.2062940

C 1.025019 1.183767 -0.7227490

H -0.435036 2.845430 -0.6813570

H -0.226120 -1.314833 -1.4689010

H 1.373192 1.357254 -1.7436050

H -1.292420 -0.567875 1.2700000

Cl -2.881062 1.842191 0.2407930

Cl 2.220422 2.385181 0.1860280

Cl 2.601560 -0.957496 -1.0602980

Cl 0.188803 -2.685587 0.3800310

Cl -2.738217 -1.282129 -0.4319640

H 1.493244 -0.270275 1.5952440

O 1.604942 -0.405673 2.5880690

H 1.303924 -1.308755 2.7254180

post-reactive complex **1**→**2**: 1,2 (C4) E1cB path

C -0.729204 -1.007744 -1.1928410

C 0.356578 -1.392151 -0.5284610

C 1.276565 -0.442033 0.1747120

C 1.018203 1.051850 -0.1227290

C -0.293557 1.347652 -0.7881370

C -1.061809 0.406360 -1.3297370

H -1.418124 -1.738371 -1.5943940

H 1.806559 1.437142 -0.7705310

H -1.951506 0.675307 -1.8837590

H 1.182614 -0.625930 1.2432580

Cl 0.663136 -3.078222 -0.2294950

Cl -3.740133 -0.990472 0.3995070

Cl -0.658018 3.033044 -1.0182600

Cl 1.228568 1.990354 1.4135640

Cl 3.014392 -0.801517 -0.2120430

H -1.872714 -0.490385 1.4754310

O -1.078755 -0.286766 2.0042140

H -1.152496 0.648579 2.2138050

*trans*-1,4,5,6-tetrachlorocyclohexa-1,3-diene (**2**)

C -0.750270 0.139526 -0.7551850

C 0.750270 -0.139521 -0.7551880

C 1.394293 0.212050 0.5469640

C 0.708844 0.174061 1.6911610

C -0.708846 -0.174065 1.6911610

C -1.394295 -0.212050 0.5469630

H 1.220475 0.379442 -1.5868570

H -1.220479 -0.379427 -1.5868580

H 1.200663 0.381490 2.6327440

H -1.200663 -0.381500 2.6327430

Cl -3.086166 -0.564611 0.4906660

Cl 3.086167 0.564600 0.4906700

Cl 1.000492 -1.906660 -1.0755860

Cl -1.000491 1.906671 -1.0755760

*trans*-1,3,4,6-tetrachlorocyclohexa-1,4-diene (**3**)

C 1.162923 0.679562 0.6209310

C 1.229540 -0.678166 0.0022740

C 0.199960 -1.285799 -0.5715610

C -1.162924 -0.679562 -0.6209310

C -1.229540 0.678165 -0.0022740

C -0.199960 1.285799 0.5715600

H 1.551073 0.658672 1.6393690

H -1.551076 -0.658674 -1.6393680

H -0.308503 2.274420 1.0005550

H 0.308503 -2.274420 -1.0005570

Cl 2.778980 -1.463338 0.0913740

Cl -2.778980 1.463339 -0.0913760

Cl -2.300918 -1.805315 0.2611380

Cl 2.300919 1.805315 -0.2611350

*trans*-1,3,5,6-tetrachlorocyclohexa-1,4-diene (**4**)

C 0.992392 0.000002 -0.3434930

C 0.192436 1.240341 -0.1010670

C -1.033676 1.255601 0.4034220

C -1.759501 -0.000002 0.7486020

C -1.033673 -1.255603 0.4034210

C 0.192439 -1.240340 -0.1010690

H 1.416918 0.000002 -1.3474160

H -2.060831 -0.000003 1.7965920

H -1.552864 -2.191941 0.5659760

H -1.552870 2.191936 0.5659770

Cl 0.996357 2.723041 -0.5286190

Cl 0.996366 -2.723038 -0.5286190

Cl -3.351022 -0.000004 -0.1544570

Cl 2.443425 0.000002 0.7622810

pre-reactive complex **1**→**5**: 1,2 (C5)

C 0.176094 1.886169 -0.216300

C -0.994394 1.216928 -0.178338

C -1.170998 -0.200638 -0.180619

C 0.050612 -0.981268 -0.490288

C 1.316816 -0.306849 0.042355

C 1.441236 1.125928 -0.468364

H 0.233201 2.949527 -0.035489

H 0.170591 -1.239878 -1.545606

H 1.299483 -0.304747 1.132523

H 1.757035 1.135922 -1.513492

Cl -2.454118 2.190989 0.038066

Cl 2.814452 1.939388 0.408117

Cl 2.777693 -1.250968 -0.439811

Cl -0.049676 -2.671410 0.299802

Cl -2.563734 -0.812201 -1.146112

H -1.615340 -0.563950 1.660488

O -1.785381 -0.807859 2.605985

H -1.396603 -1.684192 2.681959

TS **1**→**5**: 1,2 (C5)

C 0.158721 1.808297 0.2707020

C 1.234897 1.152745 -0.1439660

C 1.249239 -0.305657 -0.4935550

C -0.040907 -0.993820 -0.0981750

C -1.239081 -0.129975 -0.4433580

C -1.169212 1.129753 0.4126320

H 0.211185 2.864212 0.5058610

H -1.322992 0.131669 -1.5021550

H -1.376317 0.880200 1.4539200

H 1.509910 -0.428059 -1.5477130

Cl 2.732420 2.019296 -0.3790990

Cl -2.459772 2.300361 -0.0823070

Cl -2.777287 -1.002546 -0.0515450

Cl -0.116539 -2.553232 -1.0314960

Cl 2.642408 -1.123063 0.3795610

H -0.068044 -1.226824 1.2076500

O -0.153059 -1.328290 2.4832020

H 0.747892 -1.456813 2.7942060

post-reactive complex **1**→**5**: 1,2 (C5)

C -0.086801 1.847261 0.1128360

C 0.939449 1.309119 -0.5393330

C 0.945334 -0.072199 -1.0210730

C -0.163964 -0.800672 -0.9357420

C -1.470853 -0.219725 -0.4508170

C -1.272244 0.997871 0.4590850

H -0.057105 2.858381 0.4974720

H -2.062674 0.051210 -1.3286070

H -1.174234 0.662765 1.4915630

H 1.853533 -0.475185 -1.4462890

Cl 2.375850 2.238407 -0.8657140

Cl -2.780454 1.999425 0.4185570

Cl -2.481673 -1.424845 0.4298490

Cl -0.200050 -2.409558 -1.5839280

Cl 3.333967 -1.053348 1.2078790

H -0.063137 -1.785893 1.9602780

O 0.338082 -0.956387 2.2361710

H 1.243563 -0.981484 1.8735710

pre-reactive complex **1**→**5**: 1,4 (C3)

C 0.176094 1.886169 -0.216300

C -0.994394 1.216928 -0.178338

C -1.170998 -0.200638 -0.180619

C 0.050612 -0.981268 -0.490288

C 1.316816 -0.306849 0.042355

C 1.441236 1.125928 -0.468364

H 0.233201 2.949527 -0.035489

H 0.170591 -1.239878 -1.545606

H 1.299483 -0.304747 1.132523

H 1.757035 1.135922 -1.513492

Cl -2.454118 2.190989 0.038066

Cl 2.814452 1.939388 0.408117

Cl 2.777693 -1.250968 -0.439811

Cl -0.049676 -2.671410 0.299802

Cl -2.563734 -0.812201 -1.146112

H -1.615340 -0.563950 1.660488

O -1.785381 -0.807859 2.605985

H -1.396603 -1.684192 2.681959

TS **1**→**5**: 1,4 (C3)

C -0.200649 -1.719880 -0.1369290

C -1.364829 -1.024706 -0.3568020

C -1.426797 0.415433 -0.3997950

C -0.060638 1.082460 -0.1753480

C 1.147070 0.252228 -0.6202130

C 1.051843 -1.113494 -0.0230590

H -0.253618 -2.803257 -0.0860690

H 0.059233 1.280364 0.8886660

H 1.241396 0.238477 -1.7125380

H -1.988239 0.837305 -1.2285270

Cl -2.888790 -1.883951 -0.5258110

Cl 2.451910 -2.158513 -0.3869920

Cl 2.648101 1.128077 -0.0576230

Cl -0.064779 2.697128 -0.9749880

Cl -2.534793 1.125359 1.0582760

H 1.148529 -0.661940 1.9541720

O 1.200077 -0.257414 2.8460550

H 1.918044 0.378419 2.7700730

post-reactive complex **1**→**5**: 1,4 (C3)

C -0.853151 1.781563 0.0018450

C 0.551555 1.706375 -0.4037140

C 1.160990 0.563236 -0.7013760

C 0.419011 -0.729070 -0.5393450

C -1.101448 -0.598950 -0.6609200

C -1.617133 0.692989 -0.0690490

H -1.258608 2.729683 0.3300910

H 0.688672 -1.158314 0.4271280

H -1.404792 -0.625639 -1.7107770

H 2.215972 0.508881 -0.9321320

Cl 1.416020 3.220374 -0.4463550

Cl -3.305831 0.782500 0.3088820

Cl -1.861532 -2.057157 0.0755190

Cl 0.994789 -1.925513 -1.7676730

Cl 3.503877 -1.039152 1.2408390

H -0.599207 0.408730 2.7264930

O -0.320606 -0.504997 2.6123570

H -1.140600 -0.998082 2.5050850

pre-reactive complex **1**→**5**: 1,2 (C5)

C 0.176094 1.886169 -0.216300

C -0.994394 1.216928 -0.178338

C -1.170998 -0.200638 -0.180619

C 0.050612 -0.981268 -0.490288

C 1.316816 -0.306849 0.042355

C 1.441236 1.125928 -0.468364

H 0.233201 2.949527 -0.035489

H 0.170591 -1.239878 -1.545606

H 1.299483 -0.304747 1.132523

H 1.757035 1.135922 -1.513492

Cl -2.454118 2.190989 0.038066

Cl 2.814452 1.939388 0.408117

Cl 2.777693 -1.250968 -0.439811

Cl -0.049676 -2.671410 0.299802

Cl -2.563734 -0.812201 -1.146112

H -1.615340 -0.563950 1.660488

O -1.785381 -0.807859 2.605985

H -1.396603 -1.684192 2.681959

TS **1**→**5**: 1,2 (C5)

C 0.158721 1.808297 0.2707020

C 1.234897 1.152745 -0.1439660

C 1.249239 -0.305657 -0.4935550

C -0.040907 -0.993820 -0.0981750

C -1.239081 -0.129975 -0.4433580

C -1.169212 1.129753 0.4126320

H 0.211185 2.864212 0.5058610

H -1.322992 0.131669 -1.5021550

H -1.376317 0.880200 1.4539200

H 1.509910 -0.428059 -1.5477130

Cl 2.732420 2.019296 -0.3790990

Cl -2.459772 2.300361 -0.0823070

Cl -2.777287 -1.002546 -0.0515450

Cl -0.116539 -2.553232 -1.0314960

Cl 2.642408 -1.123063 0.3795610

H -0.068044 -1.226824 1.2076500

O -0.153059 -1.328290 2.4832020

H 0.747892 -1.456813 2.7942060

post-reactive complex **1**→**5**: 1,2 (C5)

C -0.086801 1.847261 0.1128360

C 0.939449 1.309119 -0.5393330

C 0.945334 -0.072199 -1.0210730

C -0.163964 -0.800672 -0.9357420

C -1.470853 -0.219725 -0.4508170

C -1.272244 0.997871 0.4590850

H -0.057105 2.858381 0.4974720

H -2.062674 0.051210 -1.3286070

H -1.174234 0.662765 1.4915630

H 1.853533 -0.475185 -1.4462890

Cl 2.375850 2.238407 -0.8657140

Cl -2.780454 1.999425 0.4185570

Cl -2.481673 -1.424845 0.4298490

Cl -0.200050 -2.409558 -1.5839280

Cl 3.333967 -1.053348 1.2078790

H -0.063137 -1.785893 1.9602780

O 0.338082 -0.956387 2.2361710

H 1.243563 -0.981484 1.8735710

pre-reactive complex **1**→**5**: 1,2 (C3)

C 0.176094 1.886169 -0.216300

C -0.994394 1.216928 -0.178338

C -1.170998 -0.200638 -0.180619

C 0.050612 -0.981268 -0.490288

C 1.316816 -0.306849 0.042355

C 1.441236 1.125928 -0.468364

H 0.233201 2.949527 -0.035489

H 0.170591 -1.239878 -1.545606

H 1.299483 -0.304747 1.132523

H 1.757035 1.135922 -1.513492

Cl -2.454118 2.190989 0.038066

Cl 2.814452 1.939388 0.408117

Cl 2.777693 -1.250968 -0.439811

Cl -0.049676 -2.671410 0.299802

Cl -2.563734 -0.812201 -1.146112

H -1.615340 -0.563950 1.660488

O -1.785381 -0.807859 2.605985

H -1.396603 -1.684192 2.681959

TS **1**→**5**: 1,2 (C3)

C -0.135208 -1.721922 -0.1332050

C -1.331169 -1.086092 -0.1617600

C -1.428030 0.367805 -0.4460340

C -0.096548 1.067526 -0.1318120

C 1.166853 0.322916 -0.5780820

C 1.119615 -1.071435 -0.2192940

H -0.131328 -2.805498 -0.0632220

H -0.038897 1.201410 0.9480770

H 1.484822 0.572461 -1.5905680

H -1.771674 0.579080 -1.4612000

Cl -2.829083 -1.999128 -0.1504660

Cl 2.479488 -2.044498 -0.8293230

Cl 2.625866 1.259193 0.3659000

Cl -0.118725 2.725101 -0.8354140

Cl -2.700433 1.184040 0.5979370

H 1.290543 -0.915721 1.8242270

O 1.338082 -0.643586 2.7627150

H 1.917861 0.124124 2.7353110

post-reactive complex **1**→**5**: 1,2 (C3)

C 0.694673 1.805509 -0.0331500

C 1.562774 0.796081 0.0198170

C 1.245769 -0.560636 -0.5632040

C -0.259383 -0.835566 -0.6297100

C -1.106788 0.371770 -0.8913080

C -0.649940 1.577147 -0.5659450

H 0.974436 2.801120 0.2856200

H -0.601215 -1.293661 0.2995820

H -2.123531 0.209028 -1.2210220

H 1.686002 -0.599252 -1.5630800

Cl 3.190365 1.071365 0.5434270

Cl -1.661871 2.988030 -0.7142810

Cl -3.179775 -1.213952 1.3100310

Cl -0.567734 -2.091826 -1.8943010

Cl 2.029709 -1.904125 0.3436670

H -0.357638 0.791111 2.2834300

O -0.490362 -0.112662 2.5828750

H -1.359615 -0.374231 2.2282380

*trans*-1,3,5,6-tetrachlorocyclohexa-1,3-diene (**5**)

C -1.145362 0.445918 0.3457530

C -0.025441 1.477880 0.4211990

C 1.335722 0.879118 0.5497490

C 1.552536 -0.334299 0.0403110

C 0.507947 -1.121762 -0.6128570

C -0.763255 -0.740658 -0.4800590

H -0.233082 2.187577 1.2171600

H -2.061764 0.906895 -0.0133180

H 0.782019 -2.003136 -1.1774850

H 2.125008 1.465276 1.0002730

Cl -2.067135 -1.640627 -1.1756040

Cl 3.129102 -1.065783 0.1068820

Cl -0.094822 2.467348 -1.1143560

Cl -1.519206 -0.125279 2.0294770

pre-reactive complex **1**→**6**: 1,4 (C6)

C 0.176094 1.886169 -0.216300

C -0.994394 1.216928 -0.178338

C -1.170998 -0.200638 -0.180619

C 0.050612 -0.981268 -0.490288

C 1.316816 -0.306849 0.042355

C 1.441236 1.125928 -0.468364

H 0.233201 2.949527 -0.035489

H 0.170591 -1.239878 -1.545606

H 1.299483 -0.304747 1.132523

H 1.757035 1.135922 -1.513492

Cl -2.454118 2.190989 0.038066

Cl 2.814452 1.939388 0.408117

Cl 2.777693 -1.250968 -0.439811

Cl -0.049676 -2.671410 0.299802

Cl -2.563734 -0.812201 -1.146112

H -1.615340 -0.563950 1.660488

O -1.785381 -0.807859 2.605985

H -1.396603 -1.684192 2.681959

TS **1**→**6**: 1,4 (C6)

C 0.012430 1.838403 -0.6571520

C -1.120259 1.115705 -0.3492850

C -1.169597 -0.258278 -0.0817940

C 0.032581 -1.007260 -0.5750040

C 1.306126 -0.252652 -0.1927510

C 1.309862 1.215904 -0.6198810

H -0.058224 2.893217 -0.8774340

H -0.004177 -1.212440 -1.6512440

H 1.402738 -0.306713 0.8907760

H 1.965829 1.424450 -1.4583560

Cl -2.619524 2.026329 -0.3187300

Cl 2.463226 2.086231 0.7660080

Cl 2.767966 -1.070625 -0.8556310

Cl 0.131039 -2.661026 0.1917870

Cl -2.690295 -1.128714 -0.3563330

H -0.905779 -0.349305 1.9357800

O -0.648172 -0.526525 2.8640670

H -0.332881 -1.435265 2.8424370

post-reactive complex **1**→**6**: 1,4 (C6)

C 0.214366 -1.738392 -0.9947810

C 1.420576 -1.079627 -0.4855940

C 1.447898 0.226309 -0.2019480

C 0.243242 1.091460 -0.4993100

C -1.072658 0.309091 -0.5349000

C -0.943939 -1.086348 -1.0587710

H 0.297779 -2.774689 -1.2954020

H 0.418839 1.591320 -1.4549370

H -1.511169 0.251721 0.4628640

H -1.856901 -1.574511 -1.3734570

Cl 2.818457 -2.088006 -0.3033670

Cl -3.788851 -1.485355 0.9267780

Cl -2.266213 1.241950 -1.5221420

Cl 0.063778 2.441429 0.6888130

Cl 2.895573 1.034594 0.2783330

H 0.644580 -0.891820 2.4616990

O -0.160531 -0.366979 2.5103980

H 0.147534 0.540455 2.6048150

*trans*-1,2,5,6-tetrachlorocyclohexa-1,3-diene (**6**)

C -0.729852 0.462354 -0.2866140

C -1.444167 -0.728654 0.3531300

C -0.723679 -2.028436 0.1546120

C 0.601680 -2.048437 0.0428910

C 1.369916 -0.799082 0.0256520

C 0.772607 0.392048 -0.0937190

H -1.304183 -2.940929 0.2008800

Cl 1.668243 1.853324 -0.2800920

Cl 3.091243 -0.963870 0.1021350

H 1.151325 -2.978205 -0.0257150

H -1.565287 -0.545448 1.4241820

H -0.938650 0.504728 -1.3591820

Cl -3.119807 -0.834935 -0.3015390

Cl -1.429222 1.972604 0.3962090

*trans*-1,2,5,6-tetrachlorocyclohexa-1,3-diene (**7**)

C 1.559623 0.691986 -0.3161630

C 1.559623 -0.691985 0.3161630

C 0.270238 -1.031824 0.9861090

C -0.867243 -0.480525 0.5594980

C -0.867243 0.480524 -0.5594980

C 0.270238 1.031824 -0.9861090

H 2.401581 -0.796663 0.9949200

H 2.401581 0.796663 -0.9949200

H 0.276750 1.753443 -1.7923000

H 0.276750 -1.753443 1.7923000

Cl -2.372263 0.877594 -1.3234440

Cl -2.372263 -0.877594 1.3234440

Cl 1.874966 -1.911080 -1.0068410

Cl 1.874966 1.911080 1.0068410

pre-reactive complex **2**→1,2,4-TCB

C 0.755968 0.658673 0.1814740

C -0.697046 0.255315 0.3833110

C -1.079657 -0.895403 -0.4792840

C -0.187244 -1.783653 -0.9259670

C 1.222513 -1.641743 -0.5710090

C 1.666143 -0.505053 -0.0294260

H 1.082307 1.295297 0.9999790

H -0.491318 -2.631330 -1.5265200

H 1.897528 -2.464252 -0.7699660

Cl 3.339045 -0.261026 0.3686950

Cl -2.779102 -1.078183 -0.8014250

Cl -0.882679 -0.268446 2.1288770

Cl 0.844533 1.718430 -1.3064570

H -1.361194 1.161327 0.2980610

O -2.267362 2.627868 0.4130290

H -1.943050 3.204016 -0.2851360

TS **2**→1,2,4-TCB

C 0.776901 0.616017 0.2287740

C -0.697417 0.289224 0.3676800

C -1.103757 -0.837776 -0.4923840

C -0.243042 -1.717452 -1.0214680

C 1.169440 -1.642644 -0.6684370

C 1.646780 -0.560334 -0.0471140

H 1.118776 1.186019 1.0889340

H -0.579527 -2.517497 -1.6682670

H 1.816314 -2.474841 -0.9159650

Cl 3.323002 -0.421565 0.4050510

Cl -2.821970 -1.019426 -0.7680040

Cl -0.999041 -0.199962 2.1148350

Cl 0.976305 1.783083 -1.1911210

H -1.377071 1.342712 0.2476760

O -2.090006 2.514599 0.2445100

H -1.682914 3.048386 -0.4437040

post-reactive complex **2**→1,2,4-TCB

C 0.818572 -0.254924 0.8757030

C -0.563601 -0.239612 0.7468060

C -1.197812 -1.095188 -0.1514380

C -0.443196 -1.971726 -0.9217600

C 0.939237 -1.996894 -0.8021470

C 1.552545 -1.134005 0.0955850

H 1.306228 0.431971 1.5530920

H -0.942582 -2.631906 -1.6189740

H 1.530398 -2.674109 -1.4043370

Cl 3.289896 -1.128508 0.2358390

Cl -2.922044 -1.068982 -0.3489740

Cl -1.470247 0.893071 1.7003960

Cl 1.401976 2.846600 -0.5204150

H -1.982833 1.731154 -0.9448510

O -1.387061 1.867776 -1.6869310

H -0.542051 2.137709 -1.2823640

pre-reactive complex **4**→1,2,3-TCB

C 0.830932 0.018842 0.0293690

C 0.041926 -1.217541 -0.2135350

C -1.243220 -1.261166 -0.5495280

C -2.042749 -0.018856 -0.7318130

C -1.282427 1.243673 -0.5227810

C 0.003790 1.234738 -0.1872230

H -2.573029 -0.016912 -1.6834100

H -1.825040 2.175709 -0.6211060

H -1.755912 -2.207806 -0.6670220

Cl 0.913157 -2.711398 0.0442440

Cl 0.825393 2.751090 0.0969620

Cl -3.427182 -0.053603 0.4946060

Cl 2.260461 0.052547 -1.1155280

H 1.305540 0.004762 1.1054610

O 1.871370 0.024535 2.5406960

H 2.306878 -0.806992 2.7487450

TS **4**→1,2,3-TCB

C 0.826919 0.018956 0.0363910

C 0.043829 -1.215310 -0.2242170

C -1.240257 -1.260211 -0.5668510

C -2.041058 -0.018742 -0.7444750

C -1.279919 1.243224 -0.5394880

C 0.005278 1.233069 -0.1973320

H -2.581842 -0.016857 -1.6898920

H -1.820588 2.175831 -0.6426420

H -1.750730 -2.207348 -0.6896330

Cl 0.912946 -2.711720 0.0320960

Cl 0.824282 2.752018 0.0854600

Cl -3.419169 -0.053923 0.4951880

Cl 2.286908 0.053460 -1.0686210

H 1.280998 0.003831 1.1421180

O 1.805656 0.021214 2.5395590

H 2.253723 -0.808293 2.7293190

post-reactive complex **4**→1,2,3-TCB

C -1.128461 0.282034 -0.2880780

C 0.112846 0.924370 -0.2726970

C 1.294890 0.195100 -0.2967380

C 1.244278 -1.192613 -0.3377470

C 0.021545 -1.850734 -0.3568050

C -1.155440 -1.114156 -0.3312800

H 2.174159 -1.747581 -0.3528090

H -0.031246 -2.931206 -0.3858870

H 2.252633 0.700805 -0.2784680

Cl 0.205931 2.656874 -0.1973340

Cl -2.668569 -1.965270 -0.3280040

Cl 4.809826 -0.524664 -0.1718520

Cl -2.595731 1.190820 -0.2133610

H 0.382801 -0.522521 2.2047900

O -0.301261 -0.331585 2.8554530

H -0.480971 0.607266 2.7481740

pre-reactive complex **5**→1,2,4-TCB

C -0.922200 0.056553 0.3944780

C -0.088718 1.323252 0.5148870

C 1.384769 1.098937 0.5867970

C 1.888489 0.009871 0.0034920

C 1.060773 -0.994079 -0.6611530

C -0.260269 -0.962358 -0.4674390

H -0.450529 1.925133 1.3444960

H 1.533528 -1.751883 -1.2719840

H 2.013160 1.844987 1.0544870

Cl -1.294480 -2.172622 -1.1601770

Cl 3.605246 -0.287377 -0.0098080

Cl -0.437385 2.346336 -0.9726870

Cl -1.093245 -0.664673 2.0680980

H -1.974598 0.305220 0.0900960

O -3.643432 0.770776 -0.1790760

H -3.613480 1.549007 -0.7430990

TS **5**→1,2,4-TCB

C -0.873030 0.047730 -0.0595090

C -0.083552 1.226743 -0.6161070

C 1.385100 1.169795 -0.3077000

C 1.964774 -0.016809 -0.1481610

C 1.224830 -1.278082 -0.1699770

C -0.109280 -1.239178 -0.1831270

H -0.221883 1.310588 -1.6977390

H 1.766065 -2.213065 -0.1214730

H 1.954037 2.089873 -0.3322890

Cl -1.009070 -2.718673 -0.0605440

Cl 3.687820 -0.146191 0.0880240

Cl -0.775068 2.776713 0.0313340

Cl -2.469099 -0.010567 -0.9045390

H -1.130591 0.156264 1.0510910

O -1.436943 0.129168 2.6794740

H -2.313040 -0.159984 2.9494380

post-reactive complex **5**→1,2,4-TCB

C 0.268084 -1.126634 -0.4970400

C -0.705361 -1.182807 -1.4866310

C -1.974166 -0.673247 -1.2513160

C -2.254111 -0.111809 -0.0128390

C -1.299451 -0.053647 0.9902110

C -0.031339 -0.560981 0.7391620

H -0.463855 -1.623082 -2.4454830

H -1.526200 0.388034 1.9514170

H -2.733142 -0.714137 -2.0213590

Cl 1.163622 -0.457469 1.9923510

Cl -3.842251 0.536518 0.2965810

Cl 1.068512 2.460493 -0.8529240

Cl 1.853048 -1.748072 -0.8390380

H 3.505857 0.250860 0.4594470

O 3.766730 1.121778 0.1466620

H 2.931726 1.523867 -0.1550780

pre-reactive complex **5**→1,3,5-TCB

C -0.922200 0.056553 0.3944780

C -0.088718 1.323252 0.5148870

C 1.384769 1.098937 0.5867970

C 1.888489 0.009871 0.0034920

C 1.060773 -0.994079 -0.6611530

C -0.260269 -0.962358 -0.4674390

H -0.450529 1.925133 1.3444960

H 1.533528 -1.751883 -1.2719840

H 2.013160 1.844987 1.0544870

Cl -1.294480 -2.172622 -1.1601770

Cl 3.605246 -0.287377 -0.0098080

Cl -0.437385 2.346336 -0.9726870

Cl -1.093245 -0.664673 2.0680980

H -1.974598 0.305220 0.0900960

O -3.643432 0.770776 -0.1790760

H -3.613480 1.549007 -0.7430990

TS **5**→1,3,5-TCB

C -0.932546 0.589926 0.0043930

C -0.828412 -0.900243 0.2421290

C 0.510932 -1.466739 -0.0186700

C 1.581149 -0.668557 -0.1125720

C 1.522358 0.761934 0.1567510

C 0.325302 1.347174 0.2534450

H -1.771615 1.013721 0.5499180

H 2.441775 1.316731 0.2925410

H 0.606590 -2.542076 -0.1209650

Cl 0.168487 3.040938 0.6216040

Cl 3.162287 -1.313341 -0.5016100

Cl -1.244298 -1.217223 2.0108340

Cl -1.374888 0.869716 -1.7740390

H -1.747532 -1.531364 -0.3805310

O -2.726584 -2.240118 -0.9701180

H -2.886229 -1.778567 -1.7983040

post-reactive complex **5**→1,3,5-TCB

C -1.401073 -1.211783 -0.1957800

C -2.027644 0.001210 0.0610480

C -1.402048 1.220271 -0.1712620

C -0.108494 1.198217 -0.6775670

C 0.559498 0.012756 -0.9530380

C -0.107226 -1.178198 -0.6992290

H -1.901744 -2.150545 -0.0020930

H 1.580145 0.016600 -1.3088950

H -1.904157 2.154633 0.0394200

Cl 0.718829 -2.680192 -0.9958380

Cl 0.712881 2.706985 -0.9525670

Cl -3.647577 -0.005620 0.6981000

Cl 3.489901 0.004623 0.8667060

H 0.009573 0.060888 1.8728580

O 0.742244 -0.090184 2.4768190

H 1.541564 -0.053484 1.9203120

pre-reactive complex **6**→1,2,3-TCB

C 0.652135 0.398878 -0.0743350

C 1.306039 -0.861408 -0.6229730

C 0.549392 -2.116839 -0.3124630

C -0.766518 -2.085869 -0.1111160

C -1.496597 -0.816159 -0.1004570

C -0.848921 0.354912 -0.1422750

H 1.088688 -3.054590 -0.3613290

Cl -1.685815 1.857776 0.0346290

Cl -3.223044 -0.926400 0.0477580

H -1.334325 -2.995553 0.0378160

H 1.455303 -0.790457 -1.7037710

Cl 2.998211 -0.988704 0.0361610

Cl 1.334899 1.814166 -0.9747410

H 0.922126 0.574834 1.0356990

O 1.156942 0.833455 2.6117740

H 2.027214 0.490779 2.8343820

TS **6**→1,2,3-TCB

C 0.524543 0.751476 0.3142490

C 1.441703 -0.426556 0.0250250

C 0.909693 -1.336720 -1.0302400

C -0.402626 -1.416814 -1.2656940

C -1.346971 -0.613912 -0.4873580

C -0.926893 0.396749 0.2835360

H 1.616444 -1.956024 -1.5688230

Cl -1.987986 1.408414 1.1940560

Cl -3.020506 -1.035665 -0.6206920

H -0.810907 -2.089480 -2.0092880

H 0.794485 1.221701 1.2563990

Cl 1.580529 -1.390505 1.5810400

Cl 0.823532 2.034544 -0.9526230

H 2.477406 -0.102602 -0.1735910

O 4.231642 0.179020 -0.5826130

H 5.148072 0.083500 -0.3111590

post-reactive complex **6**→1,2,3-TCB

C -0.261687 0.687457 0.7821760

C -0.853183 -0.320931 1.5305290

C -0.206051 -1.540604 1.6725420

C 1.028123 -1.754180 1.0719260

C 1.615038 -0.739317 0.3256470

C 0.979251 0.495463 0.1696840

H -0.666511 -2.330588 2.2517360

Cl 1.695877 1.743269 -0.7853430

Cl 3.152480 -1.040050 -0.4212370

H 1.541692 -2.701107 1.1744250

H -1.819463 -0.145267 1.9837300

Cl -3.886647 -0.748267 -0.6345420

Cl -1.109879 2.190393 0.6016400

H -0.469537 -1.603667 -1.1827240

O -1.001364 -1.202605 -1.8767900

H -1.865316 -1.036726 -1.4566950

pre-reactive complex **6**→1,2,4-TCB

C 0.524543 0.751476 0.3142490

C 1.441703 -0.426556 0.0250250

C 0.909693 -1.336720 -1.0302400

C -0.402626 -1.416814 -1.2656940

C -1.346971 -0.613912 -0.4873580

C -0.926893 0.396749 0.2835360

H 1.616444 -1.956024 -1.5688230

Cl -1.987986 1.408414 1.1940560

Cl -3.020506 -1.035665 -0.6206920

H -0.810907 -2.089480 -2.0092880

H 0.794485 1.221701 1.2563990

Cl 1.580529 -1.390505 1.5810400

Cl 0.823532 2.034544 -0.9526230

H 2.477406 -0.102602 -0.1735910

O 4.231642 0.179020 -0.5826130

H 5.148072 0.083500 -0.3111590

TS **6**→1,2,4-TCB

C 0.527112 0.712904 0.3030830

C 1.461384 -0.435158 -0.0067800

C 0.887236 -1.445951 -0.9151520

C -0.425879 -1.503418 -1.1856290

C -1.356216 -0.648489 -0.4576630

C -0.922621 0.372144 0.2949960

H 1.569349 -2.157049 -1.3701580

Cl -1.975991 1.386021 1.2244500

Cl -3.039974 -1.050814 -0.5802190

H -0.839626 -2.215992 -1.8879150

H 0.808854 1.203886 1.2305390

Cl 1.871805 -1.275292 1.5832070

Cl 0.773819 2.029001 -0.9815270

H 2.619430 -0.042090 -0.3972070

O 3.828490 0.324556 -0.8234140

H 4.483783 -0.208959 -0.3655670

post-reactive complex **6**→1,2,4-TCB

C 0.652665 -0.863840 0.9091200

C 1.562825 -0.925804 -0.1340720

C 1.156728 -0.884517 -1.4605370

C -0.197405 -0.777047 -1.7428210

C -1.127015 -0.707295 -0.7124170

C -0.699795 -0.750072 0.6119520

H 1.883443 -0.934133 -2.2605260

Cl -1.827249 -0.646102 1.9244740

Cl -2.807642 -0.536681 -1.1054370

H -0.539232 -0.739584 -2.7692480

H 0.982196 -0.893911 1.9393520

Cl 3.264094 -1.049402 0.2382300

Cl -0.662393 2.762727 0.0500840

H 1.518770 2.438094 -0.0395630

O 2.487647 2.368469 -0.1185100

H 2.729866 1.614005 0.4257480

pre-reactive complex **7**→1,2,4-TCB

C -1.355467 -0.324227 0.2450490

C -0.999732 1.143247 0.4112470

C 0.438446 1.384926 0.7274980

C 1.361590 0.512444 0.3188090

C 0.977753 -0.705771 -0.4167950

C -0.294816 -1.105683 -0.4527370

H -1.653627 1.612803 1.1412770

H -0.579755 -2.014120 -0.9686140

H 0.716025 2.287603 1.2558010

Cl 2.209653 -1.612210 -1.2398250

Cl 3.039580 0.791083 0.6642890

Cl -1.391140 1.993681 -1.1648830

Cl -1.569755 -1.031484 1.9260760

H -2.340049 -0.462202 -0.2379510

O -3.913142 -1.059825 -0.9605870

H -4.505838 -0.773309 -1.6604040

TS **7**→1,2,4-TCB

C -1.044599 1.023304 0.4666960

C -1.360218 -0.425733 0.1501220

C -0.209148 -1.161683 -0.4322640

C 1.028727 -0.692488 -0.4000240

C 1.342984 0.541018 0.3456130

C 0.382537 1.337222 0.7781690

H -1.696545 1.400182 1.2336260

H 0.605619 2.219381 1.3477940

H -0.410645 -2.100268 -0.9168590

Cl 3.004989 0.915464 0.7100250

Cl 2.314164 -1.547178 -1.2180480

Cl -1.742586 -1.259822 1.7855380

Cl -1.509413 2.061340 -1.0068040

H -2.505490 -0.631147 -0.5631510

O -3.538660 -0.906798 -1.1655110

H -3.666962 -0.250260 -1.8292700

post-reactive complex **7**→1,2,4-TCB

C 1.612866 -0.978338 0.0479730

C 1.029319 -1.811330 -0.8968030

C -0.354904 -1.866790 -0.9777550

C -1.139456 -1.096420 -0.1280740

C -0.534918 -0.269175 0.8151990

C 0.849039 -0.210519 0.9126680

H 1.642996 -2.406034 -1.5605110

H 1.313693 0.456166 1.6258950

H -0.833002 -2.505576 -1.7091080

Cl -1.479916 0.726124 1.8748550

Cl -2.866458 -1.161294 -0.2891660

Cl -0.526002 2.148391 -1.8267730

Cl 3.350969 -0.883300 0.1527850

H 0.902795 2.466133 -0.1603070

O 1.544589 2.704382 0.5339090

H 1.709042 3.641033 0.3945930

1,2,3-TCB

C 0.000075 2.501454 0.0000020

C 1.204012 1.811413 0.0000000

C 1.200597 0.423019 0.0000010

C -0.000009 -0.292191 -0.0000030

C -1.200555 0.423084 -0.0000040

C -1.203896 1.811483 0.0000030

H 0.000111 3.583663 0.0000050

H 2.148951 2.338394 0.0000010

H -2.148811 2.338506 0.0000070

Cl -2.724052 -0.410904 0.0000030

Cl -0.000107 -2.021021 -0.0000010

Cl 2.724065 -0.411025 -0.0000010

1,2,4-TCB

C -1.295519 -1.464675 0.0000010

C -1.715276 -0.141097 -0.0000020

C -0.807981 0.906901 -0.0000030

C 0.552349 0.619583 -0.0000030

C 0.991970 -0.703325 -0.0000010

C 0.064359 -1.738981 0.0000020

H -2.017162 -2.270641 0.0000040

H -1.144816 1.935177 -0.0000030

H 0.413999 -2.763395 0.0000050

Cl 2.683621 -1.085499 0.0000030

Cl 1.677748 1.939910 -0.0000020

Cl -3.419689 0.217849 0.0000010

1,3,5-TCB

C 1.028999 -0.908413 0.0000000

C -0.278144 -1.375881 0.0000020

C -1.300791 -0.437000 0.0000020

C -1.051995 0.928673 0.0000000

C 0.272132 1.344922 -0.0000010

C 1.330571 0.446629 -0.0000010

H -0.492549 -2.435981 0.0000020

H -1.862725 1.644419 0.0000000

H 2.355520 0.791299 -0.0000030

Cl 0.617548 3.051257 -0.0000030

Cl -2.952341 -0.990767 0.0000030

Cl 2.334507 -2.060097 0.0000000

1,3-dichlorocyclohex-2-en-1-yl localized anion

C 1.244239 0.011737 -0.5240740

C 0.016589 -0.713527 -0.3104660

C -1.138115 -0.098120 0.0241710

C -1.304620 1.381212 0.2202870

C -0.026159 2.115745 -0.2169640

C 1.242363 1.349493 0.1804830

H -1.542558 1.603447 1.2680590

H -0.037331 2.231571 -1.3050920

H 1.240405 1.226985 1.2796780

Cl -2.661085 -1.024736 0.0491120

H -2.154833 1.742976 -0.3689420

H -0.014940 3.119675 0.2148430

H 2.126759 1.941143 -0.0744840

Cl 2.671020 -0.996244 0.1401850

H 0.007822 -1.788379 -0.4727440

TS 1,3-dichlorocyclohex-2-en-1-yl delocalized anion

C 0.201076 -0.008801 1.2140850

C 0.402194 -0.643979 0.0000000

C 0.201076 -0.008801 -1.2140850

C -0.558338 1.281430 -1.2851480

C -0.297818 2.089684 0.0000000

C -0.558338 1.281430 1.2851480

H -1.644177 1.118575 -1.3989630

H 0.748216 2.412748 0.0000000

H -1.644177 1.118575 1.3989630

Cl 0.201076 -0.990796 -2.7112480

H -0.249233 1.882178 -2.1484910

H -0.916341 2.991545 0.0000000

H -0.249233 1.882178 2.1484910

Cl 0.201076 -0.990796 2.7112480

H 0.779257 -1.664523 0.0000000

1,2-dichlorocyclohex-2-en-1-yl anion

C 0.494606 -1.645166 -0.1564110

C -0.360613 -0.657447 0.1724640

C -0.025768 0.615712 0.7239440

C 1.482448 0.793286 0.8246890

C 2.238527 0.149084 -0.3472800

C 1.966287 -1.353865 -0.3326910

H 0.148609 -2.665712 -0.2809280

H 1.814178 0.307323 1.7520100

H 1.871514 0.582153 -1.2847290

H 2.542313 -1.821934 0.4768810

Cl -2.130398 -1.051660 0.0550900

Cl -0.722898 1.981779 -0.3695570

H 1.725778 1.852957 0.9265570

H 3.313526 0.341592 -0.2913040

H 2.317196 -1.818019 -1.2608390
